# Metabolic engineering of *Saccharomyces cerevisiae* for 7-dehydrocholesterol overproduction

**DOI:** 10.1186/s13068-018-1194-9

**Published:** 2018-07-16

**Authors:** Xiao-Jing Guo, Wen-Hai Xiao, Ying Wang, Ming-Dong Yao, Bo-Xuan Zeng, Hong Liu, Guang-Rong Zhao, Ying-Jin Yuan

**Affiliations:** 1 Key Laboratory of Systems Bioengineering (Ministry of Education), School of Chemical & Engineering, Tianjin University, No. 92, Weijin Road, Nankai District, Tianjin, 300072 People’s Republic of China; 2 SynBio Research Platform, Collaborative Innovation Center of Chemical Science and Engineering (Tianjin), Tianjin University, Tianjin, 300072 People’s Republic of China

**Keywords:** Metabolic engineering, 7-DHC, Host manipulation, DHCR24, *Saccharomyces cerevisiae*

## Abstract

**Background:**

7-Dehydrocholesterol (7-DHC) has attracted increasing attentions due to its great medical value and the enlarging market demand of its ultraviolet-catalyzed product vitamin D_3_. Microbial production of 7-DHC from simple carbon has been recognized as an attractive complement to the traditional sources. Even though our previous work realized 7-DHC biosynthesis in *Saccharomyces cerevisiae*, the current productivity of 7-DHC is still too low to satisfy the demand of following industrialization. As increasing the compatibility between heterologous pathway and host cell is crucial to realize microbial overproduction of natural products with complex structure and relative long pathway, in this study, combined efforts in tuning the heterologous Δ^24^-dehydrocholesterol reductase (DHCR24) and manipulating host cell were applied to promote 7-DHC accumulation.

**Results:**

In order to decouple 7-DHC production with cell growth, inducible GAL promoters was employed to control 7-DHC synthesis. Meanwhile, the precursor pool was increased via overexpressing all the mevalonate (MVA) pathway genes (*ERG10*, *ERG13*, *tHMG1*, *ERG12*, *ERG8*, *ERG19*, *IDI1*, *ERG20*). Through screening DHCR24s from eleven tested sources, it was found that DHCR24 from *Gallus gallus* (*Gg*_DHCR24) achieved the highest 7-DHC production. Then 7-DHC accumulation was increased by 27.5% through stepwise fine-tuning the transcription level of *Gg*_DHCR24 in terms of altering its induction strategy, integration position, and the used promoter. By blocking the competitive path (Δ*ERG6*) and supplementing another copy of *Gg*_DHCR24 in locus *ERG6*, 7-DHC accumulation was further enhanced by 1.07-fold. Afterward, 7-DHC production was improved by 48.3% (to 250.8 mg/L) by means of deleting *NEM1* that was involved in lipids metabolism. Eventually, 7-DHC production reached to 1.07 g/L in 5-L bioreactor, which is the highest reported microbial titer as yet known.

**Conclusions:**

Combined engineering of the pathway and the host cell was adopted in this study to boost 7-DHC output in the yeast. 7-DHC titer was stepwise improved by 26.9-fold compared with the starting strain. This work not only opens large opportunities to realize downstream de novo synthesis of other steroids, but also highlights the importance of the combinatorial engineering of heterologous pathway and host to obtain microbial overproduction of many other natural products.

**Electronic supplementary material:**

The online version of this article (10.1186/s13068-018-1194-9) contains supplementary material, which is available to authorized users.

## Background

**Fig. 1:**
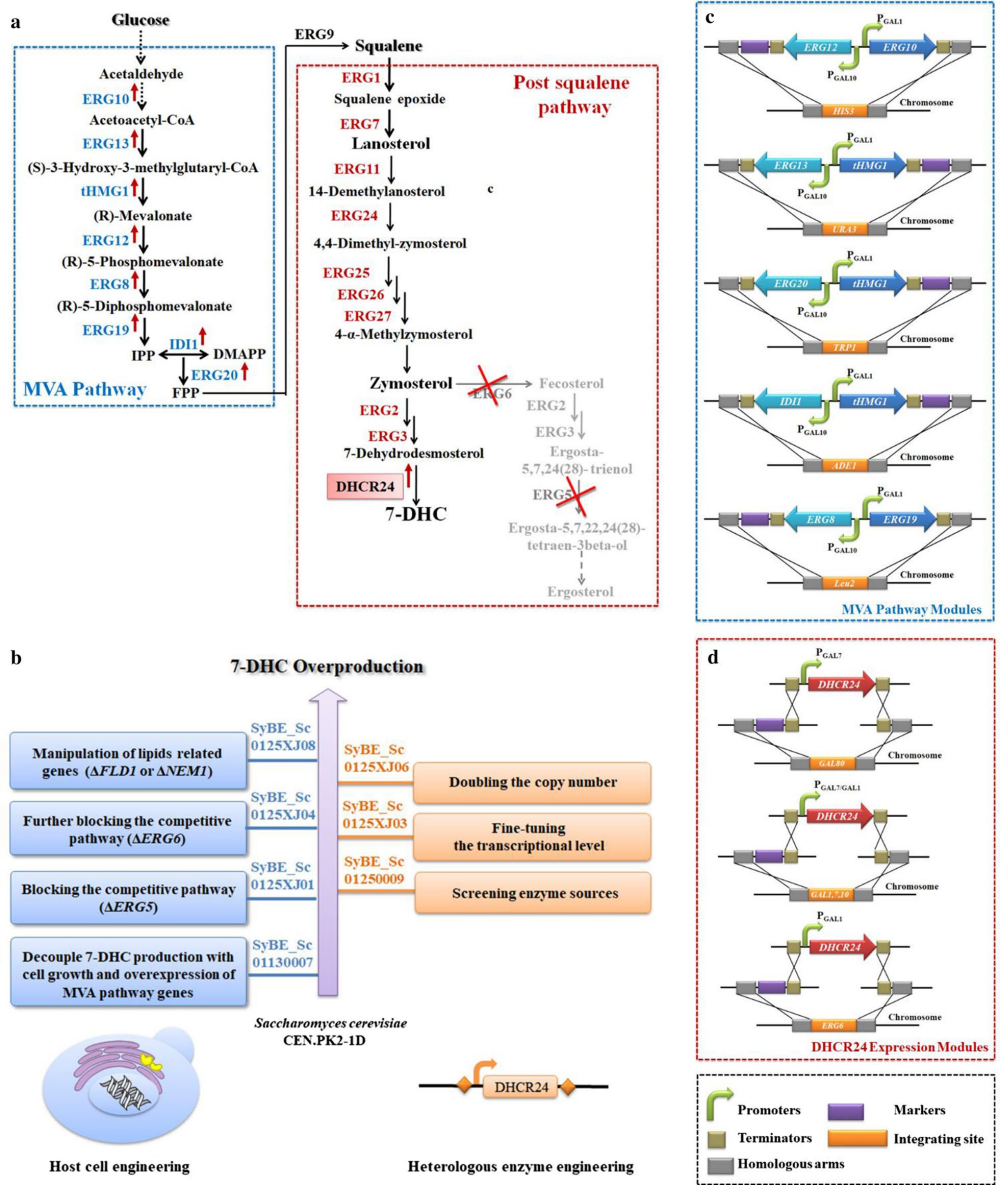
Overview of 7-DHC biosynthesis pathway and the engineering strategies applied in this study. **a** Overview of 7-DHC biosynthesis pathway in yeast. The MVA pathway is highlighted in blue and boxed, while the post-squalene pathway is highlighted in red and boxed. The blocked endogenous ergosterol synthesis pathway from zymosterol is illustrated in gray. An upward pointing arrow is used to indicate protein overexpression, and an “X” on a particular enzyme suggests that it is deleted. **b** Schematic representation of the engineering strategies to enhance 7-DHC production in *S. cerevisiae*. The host cell (blue) is engineered in combination with the only heterologous enzyme DHCR24 (orange). **c** The genetic modification for overexpressing endogenous MVA pathway genes. **d** The genetic modification for introducing *DHCR24* expression modules along with disruption of *GAL80*, *GAL7*,*10*,*1* or *ERG6*

7-Dehydrocholesterol (7-DHC) is a high-valued sterol which can be directly converted into vitamin D_3_ under ultraviolet B radiation [1]. Intake of adequate vitamin D_3_ is not only essential to maintain musculoskeletal health, but also can reduce the risk of immune disorders, cardiovascular diseases, and many types of cancers [2, 3]. Nowadays, many groups have recognized vitamin D deficiency as a worldwide public health problem, which has paved the way for a huge demand of vitamin D_3_ or its direct precursor 7-DHC every year [2, 4]. Microbial production of 7-DHC from simple carbon (such as glucose) has been recognized as an attractive complement to the traditional sources by chemical synthesis and biotransformation [5]. Through blocking the endogenous ergosterol synthesis pathway (e.g., Δ*ERG5*) along with introducing the heterologous Δ^24^-dehydrocholesterol reductase (DHCR24) (Fig. 1a), heterologous production of 7-DHC has been successfully achieved in a safe (generally recognized as safe, GRAS) and robust host *Saccharomyces cerevisiae* [5–7]. However, the highest reported 7-DHC titer is 44.49 mg/L so far [7], which is still too low to satisfy the following industrialization process.

7-DHC biosynthesis pathway covers eight genes in mevalonate (MVA) pathway and nine genes in post-squalene pathway (Fig. 1a). In order to achieve an optimal output of the targeted pathway, it is preferred to engineer the pathway modules to balance the metabolic flux among these modules. Pathway engineering in terms of enlarging the precursor pools, blocking the competitive pathway, and introducing heterologous post-squalene pathway genes (i.e., *ERG2*,*3* from *Mus musculus*), has been proven to be efficient to promote 7-DHC productivity [5–7]. However, the pathways are not isolated from the rest of cellular metabolism; in fact, they are tightly regulated by the endogenous system [8]. For instance, sterols accumulation is closely coupled to lipids synthesis in *S. cerevisiae* [9]. As reported by Fei et al. [10], sterols storage was upregulated by 70% in *FLD1* (*YLR404W*, few lipid droplets gene1) deletion strain, along with the enlarged lipid droplets. It was also reported by Park et al. [11] that the loss of *PAH1* (*YMR165C*, encoding phosphatidate phosphatase) led to striking changes in triacylglycerol and phospholipid metabolism, along with a significant increase on ergosterol synthesis. Therefore, manipulation of lipids metabolism to increase the flux flow toward sterol synthesis pathway would be helpful to improve 7-DHC accumulation in yeast. Besides that, introducing heterologous modules always upsets the original intracellular balance [12, 13]. And heterologous sterols (such as campesterol) would bring cell burden via adhering or inserting to membrane structure [14–16]. In this case, decouple the cell growth from the product synthesis by employing inducible promoter [17] or regulating lipids metabolism to improve sterol storage [18] might alleviate this cell burden. To sum up, increasing the compatibility between heterologous pathway and host cell is crucial to realize microbial overproduction of heterologous chemicals. And in addition to pathway engineering, the settlements of some metabolic and regulatory issues within hosts also offer promising approaches to enhance product output. Thus, insufficient host engineering besides modification of pathway modules might be the reason for low 7-DHC titer in the previous works [5–7].

In this article, combined efforts in manipulating host and 7-DHC synthesis pathway were conducted to promote 7-DHC output base on our previous study [7] (Fig. 1b). On the one hand, decoupling 7-DHC production with the cell growth as well as deleting lipids metabolism gene(s) were adopted to apply host engineering. On the other hand, for pathway engineering, this work would mainly focus on tuning DHCR24 via screening enzyme sources and adjusting its transcriptional level. Consequently, combinatorial engineering of the heterologous enzyme and the host cell achieved 26.9-fold enhancement on 7-DHC output (to 1.07 g/L), which highlights the importance of this combinatorial engineering strategy to improve the compatibility between heterologous pathway and host cell for microbial overproduction of desired products.

## Results and discussion

### Preparing a modified yeast beneficial for 7-DHC synthesis

In order to guarantee sufficient precursor supply, all the functional genes in MVA pathway (Fig. 1a) were overexpressed according to Westfall et al. [17], i.e., supplementing one copy of *ERG10*, *ERG13*, *ERG12*, *ERG8*, *ERG19*, *IDI1*, and *ERG20* as well as three copies of *tHMG1* (Fig. 1c). Meanwhile, to decouple 7-DHC production with cell growth, the constitutive promoters employed in the previous study were replaced by inducible GAL promoters to control the expression of the only heterologous gene (*DHCR24*) as well as the overexpressed MVA pathway genes (Fig. 1c, d), generating the strain SyBE_Sc01130007 (Table 1 and Fig. 1b). Consequently, the whole fermentation process could be divided into glucose consumption phase (before GAL induction) and ethanol consumption phase (after GAL induction).

**Table 1:** *S. cerevisiae* strains used in this study

Strain	Description	Source
CEN.PK2-1D	*MATα*, *URA3*-*52*, *TRP1*-*289*, *LEU2*-*3112*, *HIS3Δ1*, *MAL2*-*8C*, *SUC2*	EUROSCARF
SyBE_Sc01130007	CEN.PK2-1D, *LEU2::BieR*-*ERG19*-P_GAL1,10_-*ERG8*, *ADE1::tHMG1*-P_GAL1,10_-*IDI1_ADE1*, *HIS3::HIS3*-*ERG12*-P_GAL1,10_-*ERG10*, *URA3::tHMG1*- P_GAL1,10_-*ERG13*-*URA3*, *TRP1::tHMG1*- P_GAL1,10_-*ERG20*-*TPR1*, *GAL1*,*7*,*10::HphR*	This study
SyBE_Sc0125XJ01	SyBE_Sc01130007, ∆*ERG5*	This study
SyBE_Sc01250050	SyBE_Sc0125XJ01, *GAL80::*P_GAL7_-*Hs_DHCR24*-T_PGK1_-*LEU2*	This study
SyBE_Sc01250001	SyBE_Sc0125XJ01, *GAL80::*P_GAL7_- *Cg_DHCR24*-T_PGK1_-*LEU2*	This study
SyBE_Sc01250002	SyBE_Sc0125XJ01, *GAL80::*P_GAL7_- *Tg_DHCR24*-T_PGK1_-*LEU2*	This study
SyBE_Sc01250003	SyBE_Sc0125XJ01, *GAL80::*P_GAL7_-*Mm_DHCR24*-T_PGK1_-*LEU2*	This study
SyBE_Sc01250004	SyBE_Sc0125XJ01, *GAL80::*P_GAL7_- *At_DHCR24*-T_PGK1_-*LEU2*	This study
SyBE_Sc01250006	SyBE_Sc0125XJ01, *GAL80::*P_GAL7_-*Dr_DHCR24*-T_PGK1_-*LEU2*	This study
SyBE_Sc01250007	SyBE_Sc0125XJ01, *GAL80::*P_GAL7_- *Gh_DHCR24*-T_PGK1_-*LEU2*	This study
SyBE_Sc01250008	SyBE_Sc0125XJ01, *GAL80::*P_GAL7_-*Ec_DHCR24*-T_PGK1_-*LEU2*	This study
SyBE_Sc01250009	SyBE_Sc0125XJ01, *GAL80::*P_GAL7_-*Gg_DHCR24*-T_PGK1_-*LEU2*	This study
SyBE_Sc01250010	SyBE_Sc0125XJ01, *GAL80::*P_GAL7_-*Xt_DHCR24*-T_PGK1_-*LEU2*	This study
SyBE_Sc01250011	SyBE_Sc0125XJ01, *GAL80::*P_GAL7_-*Bt_DHCR24*-T_PGK1_-*LEU2*	This study
SyBE_Sc0125H001	SyBE_Sc0125XJ01, *GAL80::*P_GAL7_-*Cg DHCR24*-*6HIS*-T_PGK1_-*LEU2*	This study
SyBE_Sc0125H002	SyBE_Sc0125XJ01, *GAL80::*P_GAL7_-*Tg DHCR24*-*6HIS*-T_PGK1_-*LEU2*	This study
SyBE_Sc0125H003	SyBE_Sc0125XJ01, *GAL80::*P_GAL7_-*Mm DHCR24*-*6HIS*-T_PGK1_-*LEU2*	This study
SyBE_Sc0125H005	SyBE_Sc0125XJ01, *GAL80::*P_GAL7_-*At DHCR24*-*6HIS*-T_PGK1_-*LEU2*	This study
SyBE_Sc0125H007	SyBE_Sc0125XJ01, *GAL80::*P_GAL7_-*Dr DHCR24*-*6HIS*-T_PGK1_-*LEU2*	This study
SyBE_Sc0125H009	SyBE_Sc0125XJ01, *GAL80::*P_GAL7_-*Gh DHCR24*-*6HIS*-T_PGK1_-*LEU2*	This study
SyBE_Sc0125H010	SyBE_Sc0125XJ01, *GAL80::*P_GAL7_-*Ec DHCR24*-*6HIS*-T_PGK1_-*LEU2*	This study
SyBE_Sc0125H011	SyBE_Sc0125XJ01, *GAL80::*P_GAL7_-*Gg DHCR24*-*6HIS*-T_PGK1_-*LEU2*	This study
SyBE_Sc0125H012	SyBE_Sc0125XJ01, *GAL80::*P_GAL7_-*Xt DHCR24*-*6HIS*-T_PGK1_-*LEU2*	This study
SyBE_Sc0125H013	SyBE_Sc0125XJ01, *GAL80::*P_GAL7_-*Bt DHCR24*-*6HIS*-T_PGK1_-*LEU2*	This study
SyBE_Sc0125H050	SyBE_Sc0125XJ01, *GAL80::*P_GAL7_-*Hs DHCR24*-*6HIS*-T_PGK1_-*LEU2*	This study
SyBE_Sc0125XJ02	SyBE_Sc0125XJ01, *GAL7*,*10*,*1::*P_GAL7_-*Gg_DHCR24*-T_CYC1_-*URA3*	This study
SyBE_Sc0125XJ03	SyBE_Sc0125XJ01, *GAL7*,*10*,*1::*P_GAL1_-*Gg_DHCR24*-T_CYC1_-*URA3*	This study
SyBE_Sc0125X001	SyBE_Sc0125XJ01, *GAL7*,*10*,*1::*P_GAL1_-*Gg_DHCR24*-T_CYC1_	This study
SyBE_Sc0125XJ04	SyBE_Sc0125XJ01, *GAL7*,*10*,*1::*P_GAL1_-*Gg_DHCR24*-T_CYC1_, *ERG6::LEU2*	This study
SyBE_Sc0125XJ06	SyBE_Sc0125XJ01, *GAL7*,*10*,*1::*P_GAL1_-*Gg_DHCR24*-T_CYC1_; ∆*ERG6::*P_GAL1_-*Gg_DHCR24*-T_CYC1_-*LEU2*	This study
SyBE_Sc0125XJ07	SyBE_Sc0125XJ06, *FLD1::URA3*	This study
SyBE_Sc0125XJ08	SyBE_Sc0125XJ06, *NEM1::URA3*	This study
SyBE_Sc0125XJ09	SyBE_Sc0125XJ06, *FLD1::KanMX*, *NEM1::URA3*	This study

**Fig. 2:**
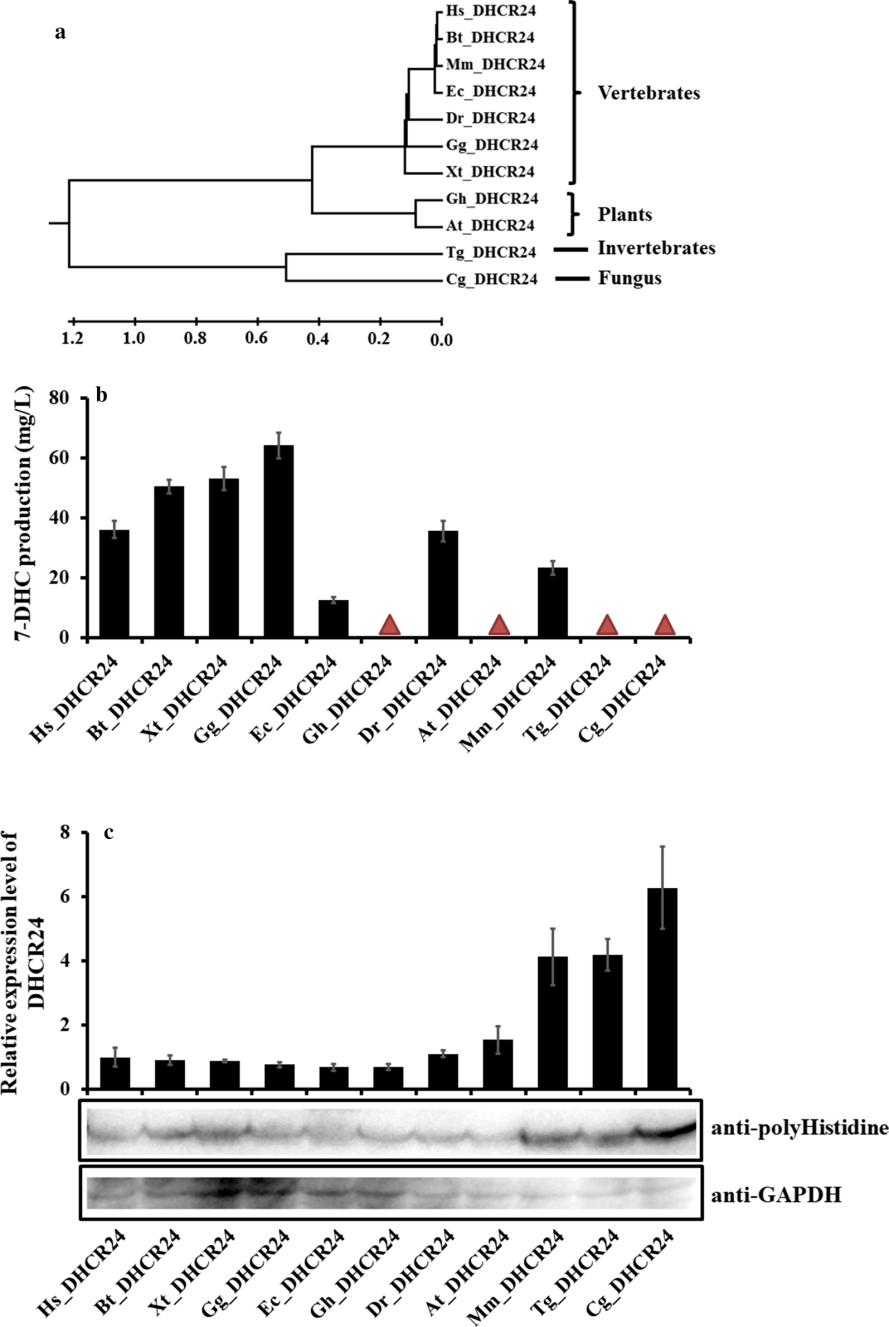
Effect of enzyme sources of DHCR24 on 7-DHC production. **a** Phylogenetic analysis of DHCR24 protein sequences selected in this study. **b** 7-DHC production in strains with DHCR24s from diversity species. Those DHCR24s that could not realize 7-DHC accumulation are denoted by red triangle. **c** Western-blotting of lysates from cells expressing polyhistidine-tag-attached DHCR24s from the selected sources. Cells were cultured in YPD medium and harvested at 40 h (ethanol consumption phase). Extracts were probed with anti-polyhistidine and anti-GAPDH (as loading control). The relative expression level of each DHCR24 is displayed as the gray scale of anti-polyhistidine band divided by that of anti-GAPDH. The error bars represent standard deviation calculated from triplicate experiments. Hs, *Homo sapiens*; Mm, *Mus musculus*; Dr, *Danio rerio*; Ec, *Equus caballus*; Gg, *Gallus gallus*; Xt, *Xenopus tropicalis*; Bt, *Bos Taurus*; At, *Arabidopsis thaliana*; Gh, *Gossypium hirsutum*; Cg, *Cryptococcus gattii*; Tg, *Trypanosoma grayi*

Moreover, our previous study has demonstrated that blocking competitive ergosterol biosynthesis pathway was essential for 7-DHC accumulation in yeast [7]. Accordingly, gene *ERG5* was knocked out to block the metabolic flux to ergosterol (Fig. 1a), obtaining strain SyBE_Sc0125XJ01 (Table 1 and Fig. 1b). Excess ergosterol can downregulate the transcription of post-squalene genes, and it was presumed that ergosterol defect would trigger sterol feedback system (such as ECM22/UPC2) [19, 20] to upregulate the genes in 7-DHC synthesis pathway [7]. Here, transcriptional analysis of strain SyBE_Sc01130007 (control) and SyBE_Sc0125XJ01 (Δ*ERG5*) revealed that disruption of *ERG5* significantly activated the transcription of all of the MVA genes (Additional file 1: Figure S1a–h) and majority of the post-squalene genes (except *ERG24*, *ERG27*, and *ERG6*, Additional file 1: Figure S1i–r) during ethanol consumption phase. The transcriptional levels of genes *ERG13*, *tHMG1*, *ERG20*, *ERG11*, *ERG25*, and *ERG3* were even enhanced during glucose consumption phase (Additional file 1: Figure S1). Notably, the transcriptions of MVA pathway genes were jointly controlled by their native promoters and GAL promoters. The upregulation efforts on these promoters were mainly represented when glucose was exhausted (Additional file 1: Figure S1), indicating a potential cross talk between galactose regulon and sterol homoeostasis. Thus, deletion of the endogenous gene *ERG5* was beneficial to 7-DHC production not only in terms of blocking the metabolic bypass but also via abolishing the suppressive effect of ergosterol on sterol synthesis pathway. And the improvement on the activities of GAL promoters by Δ*ERG5* would be beneficial for the expression of heterologous genes. Eventually, introducing DHCR24 from *Homo sapiens* (*Hs_*DHCR24) [21] generated 36.1 mg/L 7-DHC in the host SyBE_Sc0125XJ01 (Fig. 2).

### Screening DHCR24 sources

As proved in many cases, screening enzymes from diverse sources is a promising strategy to enhance the titer of desired product [22–24]. In this study, DHCR24 is the only heterologous protein which catalyzed the final step in 7-DHC synthesis pathway (Fig. 1a). So far, only three DHCR24s, which were from *H. sapiens*, *M. musculus* (*Mm_*DHCR24) [25], and *Danio rerio* (*Dr_*DHCR24) [26], have been adopted to synthesize 7-DHC [5, 6]. However, their activities have not been compared. In this study, except these three DHCR24s, four vertebrate DHCR24s from *Equus caballus* (*Ec_*DHCR24) [27], *Gallus gallus* (*Gg_*DHCR24) [28], *Xenopus tropicalis* (*Xt_*DHCR24) [29], and *Bos taurus* (*Bt_*DHCR24) [30]; two plant DHCR24s from *Arabidopsis thaliana* (*At_*DHCR24) [31] and *Gossypium hirsutum* (*Gh_*DHCR24); one invertebrate DHCR24 from *Trypanosoma grayi* (*Tg_*DHCR24) [32]; and one fungal DHCR24 from *Cryptococcus gattii* (*Cg_*DHCR24) [33] were selected (Fig. 2a and Table 2) and introduced into strain SyBE_Sc0125XJ01. As illustrated in Additional file 1: Figure S2, all the strains carrying different DHCR24s presented comparable cell growths in YPD medium. Meanwhile, none of DHCR24s from plant, invertebrate, or fungus has realized 7-DHC accumulation in yeast (Fig. 2b), even though these DHCR24s were successfully expressed in hosts (Fig. 2c). As reported, DHCR24 homologs in plants first catalyze the isomerization of the Δ^24(28)^ bond, and then deoxidize the Δ^24(25)^ bond in sterol substrate [30]. Therefore, it was speculated that plant DHCR24s require an isomeric substrate rather than 7-dehydrodesmosterol to realize the desired Δ^24^-reduction step for 7-DHC synthesis. In contrast, vertebrate DHCR24s, activities of which do not cover the initial isomerization reaction, could achieve 7-DHC synthesis in yeast at different levels (Fig. 2b). Among the seven tested vertebrate proteins, *Gg_*DHCR24 obtained the highest 7-DHC production (64.1 mg/L, Fig. 2b) which was 1.8-fold of that realized by *Hs_*DHCR24. And western-blotting assay revealed there was no statistic difference in the expression levels of DHCR24s among different vertebrate species except for *Mm_*DHCR24 (which achieved higher expression level) (Fig. 2c), suggesting *Gg_*DHCR24 might process higher enzyme activity for Δ^24^-reduction. Thus, *Gg_*DHCR24 was selected for next-step construction of 7-DHC overproducing strain. And improvements on the expression of this enzyme are probably needed to boost 7-DHC titer further.

**Table 2:** DHCR24s employed in this study

Protein	Species	Accession no.	Reaction	References
Vertebrate DHCR24s				
*HS*_DHCR24	*Homo sapiens*	Q15392	Catalyzing Δ^24^-reduction of sterol substrate	[21]
*Mm_*DHCR24	*Mus musculus*	Q8VCH6.1	Catalyzing Δ^24^-reduction of sterol substrate	[25]
*Dr_*DHCR24	*Danio rerio*	AAI65211.1	Catalyzing Δ^24^-reduction of sterol substrate	[26]
*Ec_*DHCR24	*Equus caballus*	NP_001157423.1	N.P	[27]
*Gg_*DHCR24	*Gallus gallus*	NP_001026459.1	N.P	[28]
*Xt_*DHCR24	*Xenopus tropicalis*	NP_001016800.1	N.P	[29]
*Bt_*DHCR24	*Bos taurus*	AAI50074.1	N.P	[30]
Plant DHCR24s				
*At_*DHCR24	*Arabidopsis thaliana*	Q39085.2	Catalyzing Δ^24(28)^-isomerization first, and then Δ^24^-reduction of sterol substrate	[31]
*Gh_*DHCR24	*Gossypium hirsutum*	NP_001314012	N.P	N.P
Invertebrate DHCR24				
* Tg_*DHCR24	*Trypanosoma grayi*	XP_009306481.1	N.P	[32]
Fungal DHCR24				
C*g_*DHCR24	*Cryptococcus gattii*	XP_003192961.1	N.P	[33]

*N.P* not published

### Enhancing the transcriptional level of DHCR24 via modifying its induction strategy, integration position, and used promoter

**Fig. 3:**
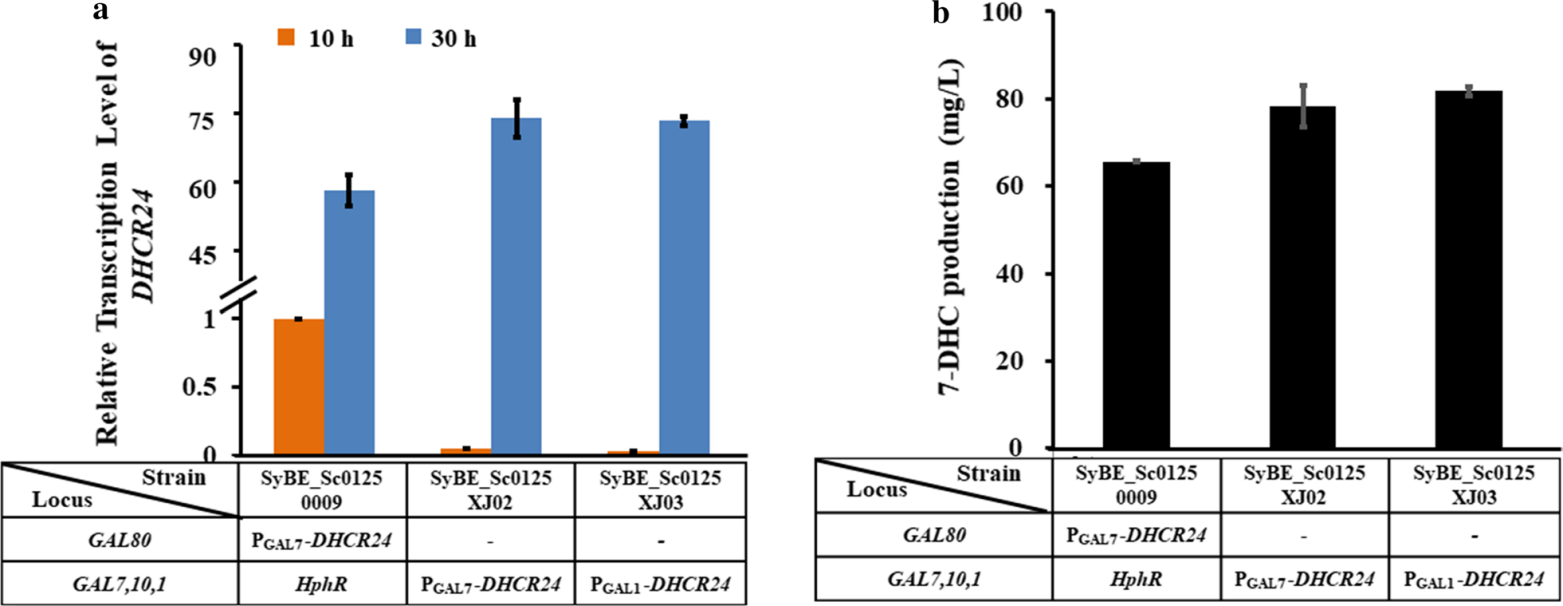
Fine-tuning the transcription level of DHCR24 to enhance 7-DHC production. **a** Real-Time PCR analysis of the transcription levels of DHCR24 in strains harvested at 10 h (orange, glucose consumption phase) and 30 h (blue, ethanol consumption phase). The relative transcription level for each gene was determined as 2^−ΔΔCt^ using gene ALG9 for normalization. All data were from at least triplicate experiments. **b** Effects of the induction strategy, genomic integration site, and promoter of DHCR24 on 7-DHC accumulation

To employ GAL promoters, *GAL7*,*1*,*10* were knocked out to eliminate galactose utilization [34]. And initially, Δ*GAL80* was applied to avoid addition of the inducer [34]. As is well known, there is another routine strategy for galactose-regulation, i.e., only deleting *GAL7*,*1*,*10* and leaving GAL80 untouched [17]. In this atudy, both these strategies were tested on the transcription levels of *DHCR24* and even 7-DHC production. In brief, in the control strain SyBE_Sc01250009, *DHCR24* expression cassette (P_GAL7_-*DHCR24*) was integrated into locus *GAL80*; whereas in strain SyBE_Sc0125XJ02, the same *DHCR24* expression cassette was inserted into locus *GAL7*,*1*,*10*, leaving a wild-type *GAL80* (Table 1). As illustrated in Fig. 3a, before galactose induction (glucose consumption phase), the basic transcription level of *DHCR24* is reduced by 94.5% in strain SyBE_Sc0125XJ02 than that in the control strain SyBE_Sc01250009, suggesting galactose regulation is stricter under wild-type *GAL80* than that under Δ*GAL80*. Correspondingly, weaker promoter leakage of *DHCR24* during glucose consumption phase would improve biomass build-up by alleviating cell toxicity brought forth by 7-DHC synthesis, which might be supported by the better cell growth of *GAL80* wild-type strain (SyBE_Sc0125XJ02 and SyBE_Sc0125XJ03) than that of Δ*GAL80* strain (SyBE_Sc01250009) (Additional file 1: Figure S3). In the meanwhile, after being activated by galactose, the transcription level of DHCR24 in strain SyBE_Sc0125XJ02 is 26.8% higher than that in the control strain (Fig. 3a). Consequently, the 7-DHC production was enhanced by 19.6% (to 78.4 mg/L) through altering the induction strategy (Fig. 3b). Besides that, it is hard to ignore the integration position effects on gene expression in *S. cerevisiae* [35]; thus, the improvement on the transcription level of *DHCR24* as well as the increase on 7-DHC production is also brought forth by the changes on the integration position of *DHCR24* expression cassette within yeast genome.

Initially, the expressions of DHCR24 were controlled by promoter GAL7 (P_GAL7_). As it was reported that the activity of promoter GAL1 (P_GAL1_) was stronger than that of P_GAL7_ [34], the promoter of *DHCR24* in strain SyBE_Sc0125XJ02 was replaced by P_GAL1_, generating strain SyBE_Sc0125XJ03 (Table 1). However, the transcription level of *DHCR24* was not increased correspondingly (Fig. 3a), indicating that promoter activity might be affected by the particular host environment. As a result, little improvement on 7-DHC (from 78.4 to 81.7 mg/L) was detected by comparing that in strain SyBE_Sc0125XJ03 with strain SyBE_Sc0125XJ02 (Fig. 3b). Despite this, since strain SyBE_Sc0125XJ03 achieve higher 7-DHC titer, this strain was still chosen for the next step of optimization. Worthy to be noticed, there was positive association between 7-DHC accumulation and *DHCR24* transcriptional level (Fig. 3). Thus, further improving the transcription level of *DHCR24*, such as doubling its copy number, might be a promising approach to enhance 7-DHC production.

### Further blocking sterol competitive pathway by Δ*ERG6*

**Fig. 4:**
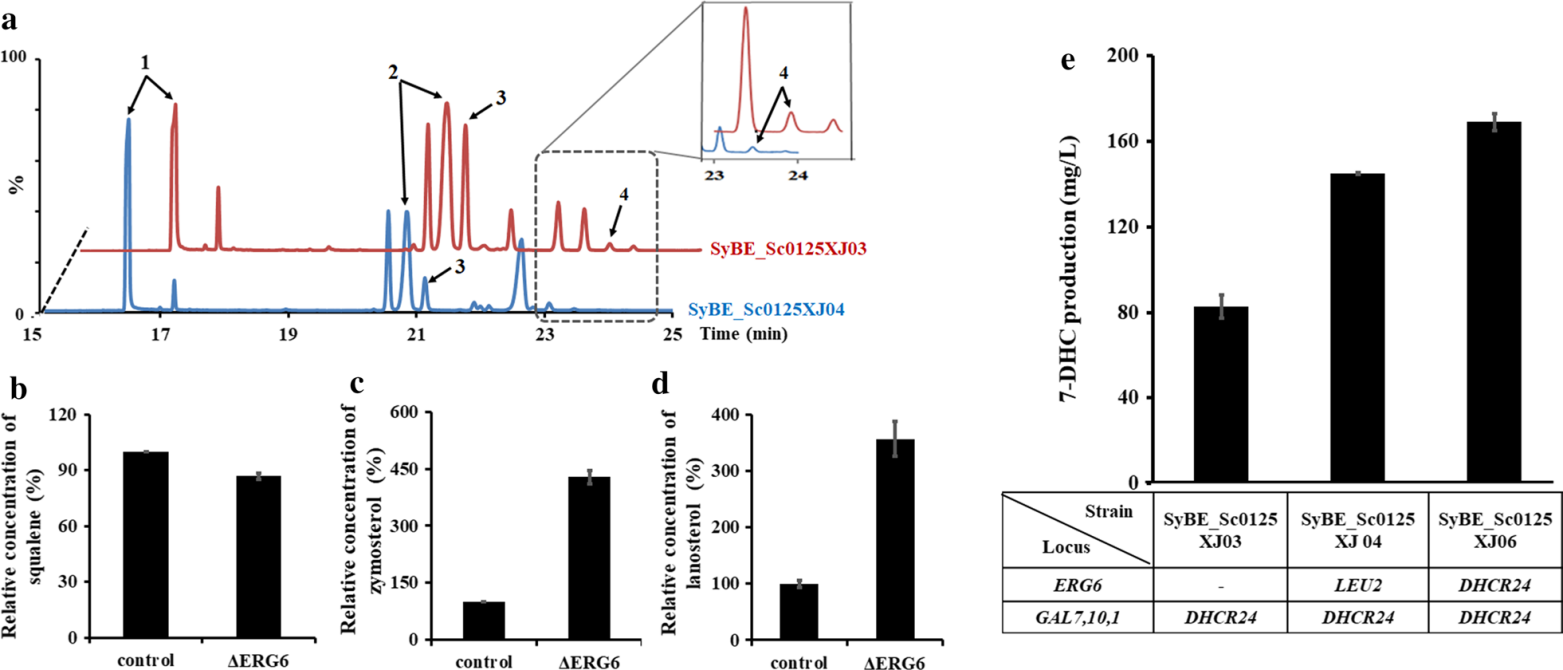
Effect of Δ*ERG6* on 7-DHC production. **a** GC/TOF-MS analysis of the fermentation products of strains SyBE_Sc0125XJ03 (red) and SyBE_Sc0125XJ04 (blue). I, squalene; II, 7-DHC; III, zymosterol; IV, lanosterol. The relative accumulations of squalene (**b**) zymosterol (**c**), and lanosterol (**d**) in strains SyBE_Sc0125XJ03 (control) and SyBE_Sc0125XJ04 (Δ*ERG6*) were determined using those in the control strain for normalization. **e** Improvement of 7-DHC accumulation via blocking the competitive path (Δ*ERG6*) and supplementing another copy of DHCR24 in locus *ERG6*. The error bars represent standard deviation calculated from triplicate experiments

In our current sterol biosynthesis pathway, there was still ERG6 existing to convert zymosterol to fecosterol, which is not required for 7-DHC synthesis. To block this by-path, gene *ERG6* was knocked out in strain SyBE_Sc0125XJ03 by marker *LEU2*, gaining strain SyBE_Sc0125XJ04 (Table 1 and Fig. 1b). As a result, deletion of *ERG6* enhanced the accumulation of its substrate zymosterol (Fig. 4a, c). Besides that, this approach also reduced the accumulation of squalene (Fig. 4b) as well as increasing the accumulation of lanosterol (Fig. 4d), indicating enlargement of the metabolic flow through post-squalene pathway. Consequently, 7-DHC production was increased by 77.6% (to 145.1 mg/L) by Δ*ERG6* (Fig. 4e). Then, adding another copy of *DHCR24* expression cassette (P_GAL1_-*DHCR24*) into locus *ERG6* (obtaining strain SyBE_Sc0125XJ06, Table 1 and Fig. 1b) further improved 7-DHC titer by 16.5% (to 169.1 mg/L, Fig. 4e). Strains SyBE_Sc0125XJ03, SyBE_Sc0125XJ04, and SyBE_Sc0125XJ06 demonstrated comparable cell growths in YPD medium (Additional file 1: Figure S4). Therefore, strain SyBE_Sc0125XJ06 was employed for further engineering.

### Engineering lipids metabolism genes

**Fig. 5:**
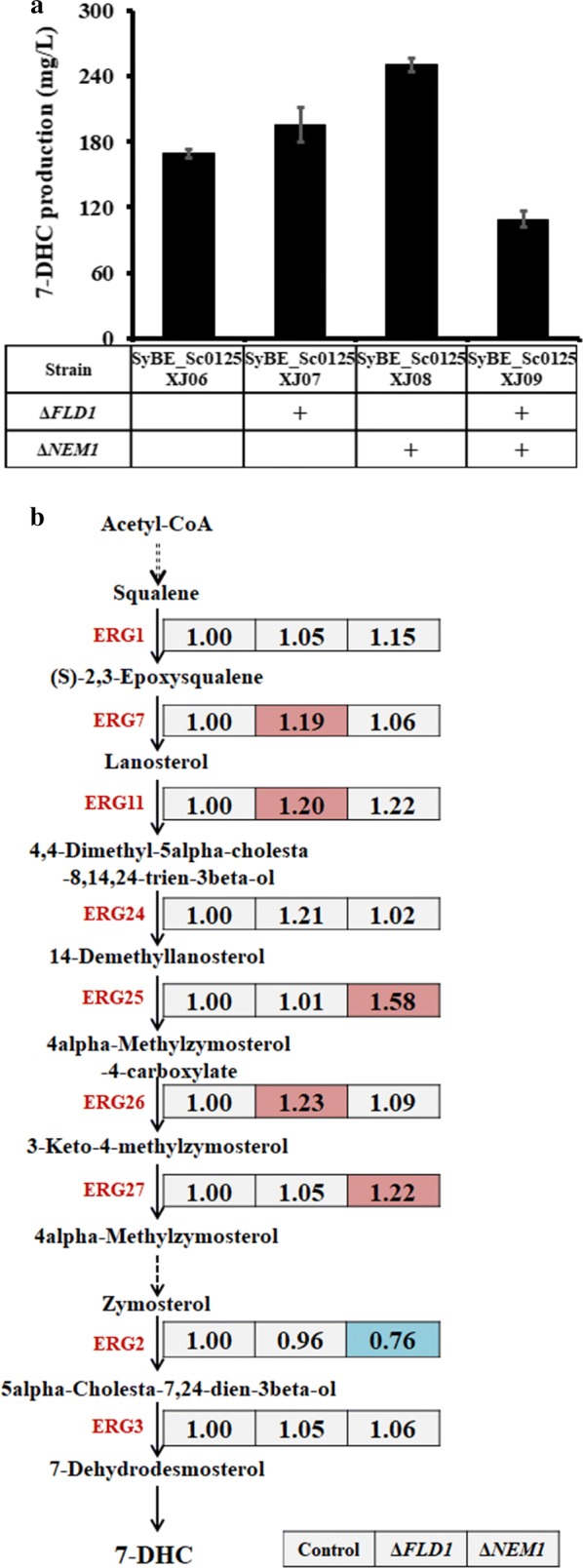
Effect of deleting lipids metabolism associated genes on 7-DHC production. **a** 7-DHC production in the control (SyBE_Sc0125XJ06) and strains with individual deletion of *FLD1* and *NEM1.* **b** Relative transcription levels of the post-squalene pathway genes in strains SyBE_Sc0125XJ06 (control), SyBE_Sc0125XJ07 (Δ*FLD1*), and SyBE_Sc0125XJ08 (Δ*NEM1*). Cells were harvested at 30 h (ethanol consumption phase). The relative transcription level for each gene was quantified by Real-Time PCR and determined as 2^−ΔΔCt^ using gene ALG9 for normalization (data listed in the box). All data were from at least triplicate experiments. Significance levels of *t* test were determined for *P* < 0.05. Upregulated, downregulated genes, and genes without significant transcriptional difference are denoted in red, blue, and gray, respectively

As described above, endogenous sterol accumulation was enhanced by Δ*FLD1* [10] as well as by Δ*PAH1* [11]. Meanwhile, NEM1 (YHR004C) is the catalytic subunit of NEM1-SPO7 phosphatase, which is responsible for dephosphorylation of PAH1 to activate its function [36]. Therefore, besides deletion of *FLD1*, knocking out *NEM1* would also be benefit for 7-DHC production. Accordingly, these two genes were individually knocked out in strain SyBE_Sc0125XJ06, generating strains SyBE_Sc0125XJ07 (Δ*FLD1*) and SyBE_Sc0125XJ08 (Δ*NEM1*), respectively (Table 1). As illustrated in Fig. 5a, using strain SyBE_Sc0125XJ06 as the control, Δ*FLD1* and Δ*NEM1* achieved 15.7% (to 195.7 mg/L) and 48.3% (to 250.8 mg/L) improvement on 7-DHC production, respectively. However, further combination of Δ*FLD1*/Δ*NEM* reduced the 7-DHC titer to 109.0 mg/L (Fig. 5a). Therefore, *NEM1*-deleted strain SyBE_Sc0125XJ08 was employed in further fed-batch fermentation. Besides, 7-DHC titer of strain SyBE_Sc0125XJ08 in YPD medium was 8.25-fold higher than that achieved in SC medium with the same glucose concentration, while the biomass (OD_600_) under YPD medium when harvested was only 1.25-fold higher than that under SC medium (Additional file 1: Figure S5). These data demonstrated the unknown effect of complex media on the 7-DHC titers besides boosting cell growth.

**Fig. 6:**
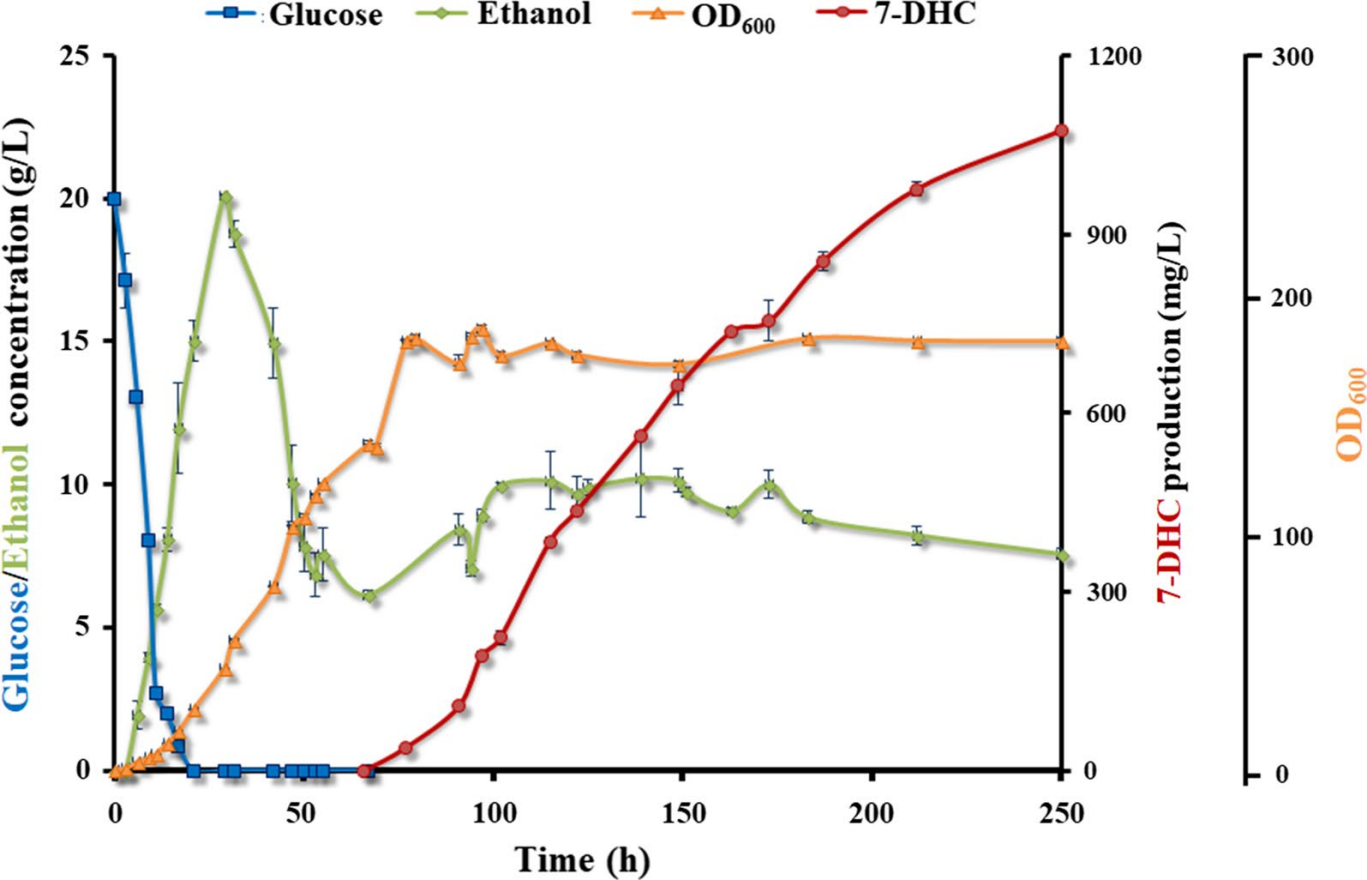
7-DHC production in fed-batch fermentation. Profile 7-DHC titer (red), glucose (blue), ethanol (green), and biomass (orange) during fermentation with strain SyBE_Sc0125XJ08. The error bars represent standard deviation calculated from duplicate experiments

Deletion of Δ*FLD1* or Δ*NEM1* both demonstrated modification on the profile of cellular lipids, including triacylglycerols, sterols and phospholipids, which are all compositions of cell membrane [10, 11]. Altering membrane lipid composition is a crucial for microbial stress adaptation [37]. However, neither deletion of *FLD1* nor *NEM1* could improve cell growth of 7-DHC synthesis strain in YPD medium (Additional file 1: Figure S5a), suggesting their positive effect on 7-DHC output might not due to improvement on bacterial stress adaptation by altering membrane compositions. Further, the effect of losing *FLD1* or Δ*NEM1* upon 7-DHC synthesis was investigated on transcriptional level. As shown in Fig. 5b, deletion of *FLD1* significantly upregulated post-squalene genes *ERG7*, *ERG11,* and *ERG26*. Overexpression of *ERG11* resulted in increase of downstream sterols (such as 4,4-dimethylzymosterol, zymosterol and ergosterol) in ergosterol synthetic yeast [38], which might be the reason for the increased 7-DHC productivity by Δ*FLD1*. Meanwhile, as reported by Arendt et al. [39], Δ*PAH1* stimulated a dramatic expansion of the endoplasmic reticulum (ER), which resulted in overproduction of triterpenoids (e.g., β-amyrin and its derivatives) probably by functional overproduction of ER-localized proteins. However, among those ER-located genes (*ERG1*, *ERG11*, *ERG24*-*27*, and *ERG2*-*3*) [40], only *ERG25* and *ERG27* was significantly activated by Δ*NEM1*, indicating that the enhanced 7-DHC productivity by Δ*NEM1* might not bring by ER-engineering effect. Thus, it is probably required further global transcription analysis by RNA-sequencing to expose its functional mechanism toward 7-DHC synthesis.

### Fed-batch fermentation

Fed-batch fermentation of strain SyBE_Sc0125XJ08 was carried out in 5-L bioreactor under glucose restriction strategy. Glucose concentration was controlled below 5 g/L for reducing the ethanol and glycerol produced during the fermentation process (Fig. 6). Air flow and dissolved oxygen were controlled appropriately as sterols synthesis in *S. cerevisiae* was an oxygen consumption process and too much oxygen supply would inhibit sterol synthesis [41]. As the usage of GAL promoters to control 7-DHC synthesis, the whole process was divided into cell growth stage and 7-DHC accumulation stage. In the first stage, glucose was used as the solo carbon source for biomass growth. In the second stage, galactose was added into the bioreactors, and glucose was not further supplied. During that time, ethanol was the carbon source mainly for 7-DHC accumulation. Eventually, after 250-h cultivation, 7-DHC production reached 1.07 g/L (Fig. 6), which is the highest reported microbial titer as yet known.

However, 7-DHC synthesis could be further enhanced via host engineering in yeast. On the one hand, ergosterol is essential to maintain a normal structure and function of cellular membranes [42], and ergosterol defect could also trigger redox imbalance [7]. Therefore, besides ergosterol supplement, introducing cofactor regeneration modules and building gene genetic to restrict transcription of *ERG5*-*6* only in cell growth stage would compensate for the necessary block of ergosterol biosynthesis during 7-DHC accumulation period. One the other hand, sterols stored in *S. cerevisiae* in their esterified forms, and overexpression of two endogenous sterol acyltransferases (ARE1 and ARE2) could promotes sterols accumulation [43, 44]. However, a DSM patent revealed that reducing or abolishing the activity of ARE1 or ARE2 was beneficial to 7-DHC production in yeast [45]. Even though it is hard to explain the contrary results of these works, these data suggested that modifying formation and hydrolysis of sterol esters would be another promising approach to boost 7-DHC output in future study.

## Conclusions

In this work, combined engineering of the host cell and the heterologous enzyme DHCR24 significantly improved 7-DHC productivity in *S. cerevisiae*. A modified host cell was constructed to appeal to the increased 7-DHC accumulation via decoupling 7-DHC production with cell growth, enhancing MVA pools, totally blocking the competitive path (Δ*ERG5*,*6*), as well as deleting lipids metabolism gene (Δ*NEM1*). In the meanwhile, the optimal DHCR24 sources (*Gg*_DHCR24) were obtained by screening the enzymes from diversity species. And through fine-tuning the transcription level of *Gg*_DHCR24 in terms of adjusting its induction strategy (Δ*GAL7*,*1*,*10*), integration position (loci *GAL7*,*1*,*10*, and *ERG6*), used promoter type (P_GAL1_), and copy numbers, 7-DHC production were stepwise improved accordingly. Eventually, the highest 7-DHC titer, so far known (1.01 g/L), was achieved in 5-L bioreactor, which is 26.9-fold higher than that of the starting strain. This work not only opens large opportunities to realizes downstream the de novo synthesis of other steroids, but also highlights the importance of the combinatorial engineering of heterologous pathway and host to obtain microbial overproduction of many other natural products.

## Methods

### Strains and media

*Escherichia coli* DH5α, which was used for plasmids construction, was cultivated at 37 °C in Luria–Bertani (LB) medium supplemented with 50 μg/mL kanamycin. The yeast strains used in this study were derived from *S. cerevisiae* CEN.PK2-1D and summarized in Table 1. Recombinational yeasts were selected on solid synthetic complete (SC) medium lacking appropriate nutrient component [46]. Shake flask fermentation of engineered strains was performed in modified YPD medium (2% peptone, 1% yeast extract, 4% glucose and 1% d-(+)-galactose) at 30 °C.

### Protein analysis

The protein sequences of the selected DHCR24s from *H. sapiens*, *M. musculus*, *D. rerio*, *E. caballus*, *G. gallus*, *X. tropicalis*, *B. taurus*, *A. thaliana*, *G. hirsutum*, *C. gattii*, *T. grayi* were obtained from NCBI database (https://www.ncbi.nlm.nih.gov/, Table 2). Protein sequences alignment and phylogenetic tree construction were carried out with MEGA7 [47].

### Construction of plasmids and strains

Yeast homologous recombination was applied to knock-out genes as well as to integrate genes expression cassettes. All the primers used in this study were synthesized by Genewiz Inc. (China) and listed in Additional file 1: Table S1. All the auxotroph markers, promoters, and terminators adopted here were obtained from our module library SynbioML (http://synbioml.org/). Heterologous *DHCR24* genes were codon-optimized (Additional file 1: Table S2) and synthesized by GenScript Inc. (China). All the endogenous genes involved in this study were PCR amplified from the genomic DNA of *S. cerevisiae* CEN.PK2-1D. These PCR products shared 40-bp ends homologous to the adjacent fragments or linearized vector; therefore, MVA pathway-enhancing cassettes (Fig. 1c) can be constructed by Gibson assembly method [48]. In the meanwhile, homologous arm cassettes and *DHCR24* expression cassettes (Fig. 1d) were assembled by overlap extension PCR (OE-PCR). The assembled products were cloned into plasmid pRS425K (Additional file 1: Table S3). Before yeast transformation via the LiAc/SS carrier DNA/PEG method [46], these plasmids should be treated by enzyme(s) digestion.

### Shake-flask and fed-batch fermentation

For shake flask fermentation, glycerol-stock yeasts were rejuvenated on solid YPD plate [46]. Then a single colony was picked up and inoculated into 5 mL YPD medium for overnight cultivation at 30 °C. When cells entered mid-exponential phase, the culture was transferred into 50 mL modified YPD medium or SC medium with an initial OD_600_ of 0.2. The initial glucose concentration in either YPD medium or SC medium was 40 g/L. Before cultivation, 10 g/L d-galactose was supplemented into the media to induce the gene expressions controlled by GAL promoters. Yeast cells were harvested after 100-h growth.

Fed-batch fermentation was conducted in YPD medium supplemented with 20 g/L glucose as the initial carbon source. Seed cultures were obtained via overnight culture from an OD_600_ of 0.2–8.0 in YPD medium. Then 200 mL seed cultures were transferred into a 5-L bioreactor (BLBIO-5GJG-2, Shanghai, China) with an initial OD600 of 0.8. Fermentation was performed in 2-L cultures at 30 °C. PH and air flow were controlled at 5.8 and 1 vvm, respectively. The dissolved oxygen (DO) was maintained around 40% through adjusting the agitation speed. 50% (v/v) glucose was fed periodically into the culture to keep the glucose concentration under 2 g/L. When cells entered post-log phase (OD_600_ around 140), glucose feeding was stopped, and galactose solution was added into the bioreactor with a final concentration of 20 g/L. At the same time, ethanol was fed into the culture until the end of the fermentation. At least independent duplicate samples were collected to determine the cell density, glucose concentration, ethanol concentration, and 7-DHC production [7].

### Protein expression quantification

In order to determine the expression levels of DHCR24s employed in this study, polyhistidine-tag was attached to the C-terminal of each tested DHCR24 in its particular expression cassette by PCR amplification with the primers listed in Table S1. Then the cassette was integrated into chromosome of strain syBE_Sc0125XJ01 with the same procedure to generate strain SyBE_Sc01250050 (obtaining strain SyBE_Sc0125H001-03/05/07/09/10-13/50, Table 1). These strains were grown in YPD medium for 40 h (ethanol consumption phase). The protein extraction and western-blot were then conducted according to Kinzurik et al. [49] and Rodriguez-Escudero et al. [50]. To be specific, 0.5 mL cells (OD_600_ around 20) were harvested and resuspended in 200 μL 0.1 M NaOH for 5 min incubation at room temperature. Then cell pellets were harvested, resuspended in 50 μL SDS sample buffer (60 mM Tris–HCl (pH 6.8), 5% glycerol, 2% SDS, 4% β-mercaptoethanol, 0.0025% bromophenol blue), and boiled for 10 min. 20 μL cell lysis were loaded onto 10% SDS-PAGE gel. After electrophorescence, proteins were transferred to PVDF membranes. Membranes were blocked with 5% BSA in TBST buffer (10 mM Tris (pH 8.0), 150 mM NaCl, 0.05% Tween 20), then incubated with primary anti-polyhistidine (1:2000, Rayantibody RM1001, China), or anti-GAPDH (HRP) (1:5000, Abcam ab9385, UK) overnight at 4 °C with shaking. Afterward, membranes were repeatedly washed with TBST buffer. The membrane probed to anti-polyhistidine required further incubation with secondary HRP-conjugated goat anti-mouse antibody (Rayantibody, China). Signals were detected following the of SuperSignal™ West Pico PLUS Chemiluminescent Substrate Kit (Thermo, USA) by using Azure Biosystems C280 Chemiluminescent Blot Imaging System (USA). The intensities of the bands in western-blot pictures were quantified with Quantity One (Bio-rad, USA). The relative expression level of each DHCR24 was determined as the gray scale of anti-polyhistidine band divided by that of anti-GAPDH.

### Extraction and analysis of sterols

Extraction and analysis of sterols were applied according to Su et al. [7] with some modification. Yeast cells were harvested by 12,000 rpm centrifugation and resuspended by 3 N HCl. The suspension was boiled for 5 min, and then the cells debris was washed by distilled water until pH was neutral. Just in case, NaOH solution was used to neutralize the residual HCl. The cell pellet was resuspended by 1.5 M NaOH-methonal solution and incubated at 60 °C for 4 h. Then n-hexane was added for sterols extraction with vortex. After centrifugation, the n-hexane phase was collected and dried by centrifugal vacuum evaporator. Derivatization of the dried products was conducted with *N*-methyl-*N*-(trimethylsilyl) trifluoroacetamide (MSTFA) at 30 °C for 2 h to gain the sample ready for analysis.

The sterols were separated on an Agilent 6890 gas chromatograph (GC) (USA) coupled to Waters time-of-flight mass spectrometry (TOF–MS) (USA). The gas chromatograph was equipped with a DB-5 fused-silica capillary column (30 m × 0.25 mm i.d., film thickness 0.25 μm, J&W Scientific, CA). Ions were generated by a 70 eV electron beam in EI mode at an ionization current of 40 μA. Mass spectra were acquired in a range of 50–800 *m/z*. The ion source temperature was 250 °C, and the injection site temperature was 260 °C. The temperature was initially 70 °C for 2 min, then it was increased at 30 °C/min to 250 °C, and finally followed by an increase to 280 °C at 10 °C/min. 280 °C was kept for 15 min, and was increased to the final temperature 290 °C at 5 °C/min. The final temperature was maintained for 5 min. Sterol standards (squalene, lanosterol, zymosterol, and 7-DHC) were purchased from Sigma-Aldrich (USA).

### Genes transcriptional analysis

Transcription levels of genes in 7-DHC biosynthesis pathway were analyzed by Real-Time PCR. Strains were cultured in shake flask for 10 h (glucose consumption phase) and 30 h (ethanol consumption phase), respectively, and then harvested. Total RNA extraction, reverse transcription, and quantitative PCR were carried out by Apexbio Inc. (China) based on Wang et al. [51]. The relative transcription level for each gene was determined by 2^−ΔΔCt^ method [52]. Gene *ALG9* was used for normalization [53]. All data were from at least triplicate experiments. The statistical analysis (*T* test) was conducted using the SPSS 19.0 package to demonstrate variations between the tested groups. The level of significance was set at *P* < 0.05.

## Additional file

**Additional file 1: Table S1.** Oligonucleotides used in this study. **Table S2.** The Codon-optimized sequences of DHCR24s involved in this study. **Table S3** Plasmids used in this study. **Figure S1.** Relative transcription level of the MVA pathway genes (a–h) and the post squalene pathway genes (i–r) in control and Δ*ERG5* strain. Cells were harvested at 10 h (glucose consumption phase) and 30 h (ethanol consumption phase). The relative transcription level for each gene was determined as 2^−ΔΔCt^ using gene ALG9 for normalization. All data were from at least triplicate experiments. Significance levels of t-test were determined as “*” is for P < 0.05 and “**” is for P < 0.01. **Figure S2.** Cell growths of strains with DHCR24s from diversity species in YPD medium. The error bars represent standard deviation calculated from triplicate experiments. Hs, *Homo sapiens*; Mm, *Mus musculus*; Dr, *Danio rerio*; Ec, *Equus caballus*; Gg, *Gallus gallus*; Xt, *Xenopus tropicalis*; Bt, *Bos Taurus*; At, *Arabidopsis thaliana*; Gh, *Gossypium hirsutum*; Cg, *Cryptococcus gattii*; Tg, *Trypanosoma grayi*. **Figure S3.** Cell growths of strains SyBE_Sc01250009, SyBE_Sc0125XJ02 and SyBE_Sc0125XJ03 in YPD medium. **Figure S4.** Cell growths of strains SyBE_Sc0125XJ03, SyBE_Sc0125XJ04 and SyBE_Sc0125XJ06 in YPD medium. **Figure S5.** Effect of deleting lipids metabolism associated gene(s) on biomass building-up and 7-DHC production. **a** Cell growths of strains SyBE_Sc0125XJ06, SyBE_Sc0125XJ07, SyBE_Sc0125XJ08 and SyBE_Sc0125XJ09 in YPD medium as well as that of SyBE_Sc0125XJ08 in SC medium. **b** 7-DHC production of strain SyBE_Sc0125XJ08 in YPD medium and SC medium.

## Abbreviations

7-DHC: 7-dehydrocholesterol
Hs: *Homo sapiens*
Mm: *Mus musculus*
Dr: *Danio rerio*
Ec: *Equus caballus*
Gg: *Gallus gallus*
Xt: *Xenopus tropicalis*
Bt: *Bos taurus*
At: *Arabidopsis thaliana*
Gh: *Gossypium hirsutum*
Cg: *Cryptococcus gattii*
Tg: *Trypanosoma grayi*
OE-PCR: overlap extension PCR
MVA: mevalonate
MSTFA: *N*-methyl-*N*-(trimethylsilyl) trifluoroacetamide
GC-TOF–MS: gas chromatography time-of-flight mass spectrometry
DHCR24: Δ^24^-dehydrocholesterol reductase
*FLD1*: few lipid droplets gene1
*PAH1*: phosphatidate phosphatase
DO: dissolved oxygen
