# Supplementary material for: Metabolic engineering of Saccharomyces cerevisiae for 7-dehydrocholesterol overproduction
Source: Biotechnol Biofuels. 2018 Jul 16;11:192. doi: 10.1186/s13068-018-1194-9 (PMC6047132; doi:10.1186/s13068-018-1194-9)
Supplement: Supplementary file 1 — Additional file 1: Table S1. Oligonucleotides used in this study. Table S2. The Codon-optimized sequences of DHCR24s involved in this study. Table S3 Plasmids used in this study. Figure S1. Relative transcription level of the MVA pathway genes (a–h) and the post squalene pathway genes (i–r) in control and ΔERG5 strain. Cells were harvested at 10 h (glucose consumption phase) and 30 h (ethanol consumption phase). The relative transcription level for each gene was determined as 2−ΔΔCt using gene ALG9 for normalization. All data were from at least triplicate experiments. Significance levels of t-test were determined as “*” is for P < 0.05 and “**” is for P < 0.01. Figure S2. Cell growths of strains with DHCR24s from diversity species in YPD medium. The error bars represent standard deviation calculated from triplicate experiments. Hs, Homo sapiens; Mm, Mus musculus; Dr, Danio rerio; Ec, Equus caballus; Gg, Gallus gallus; Xt, Xenopus tropicalis; Bt, Bos Taurus; At, Arabidopsis thaliana; Gh, Gossypium hirsutum; Cg, Cryptococcus gattii; Tg, Trypanosoma grayi. Figure S3. Cell growths of strains SyBE_Sc01250009, SyBE_Sc0125XJ02 and SyBE_Sc0125XJ03 in YPD medium. Figure S4. Cell growths of strains SyBE_Sc0125XJ03, SyBE_Sc0125XJ04 and SyBE_Sc0125XJ06 in YPD medium. Figure S5. Effect of deleting lipids metabolism associated gene(s) on biomass building-up and 7-DHC production. a Cell growths of strains SyBE_Sc0125XJ06, SyBE_Sc0125XJ07, SyBE_Sc0125XJ08 and SyBE_Sc0125XJ09 in YPD medium as well as that of SyBE_Sc0125XJ08 in SC medium. b 7-DHC production of strain SyBE_Sc0125XJ08 in YPD medium and SC medium. [file 13068_2018_1194_MOESM1_ESM.docx]

**Additional file 1**

**Metabolic engineering of *Saccharomyces cerevisiae* for 7-dehydrocholesterol overproduction**

Xiao-Jing Guo^1,2^, Wen-Hai Xiao^1,2^_,_ Ying Wang^1,2^_,_ Ming-Dong Yao^1,2^, Bo-Xuan Zeng^1,2^, Hong Liu^1,2^, Guang-Rong Zhao^1,2^, Ying-Jin Yuan ^1,2*^

^1^ Key Laboratory of Systems Bioengineering (Ministry of Education), School of Chemical & Engineering, Tianjin University, Tianjin, 300072, P.R. China

^2^ SynBio Research Platform, Collaborative Innovation Center of Chemical Science and Engineering (Tianjin), Tianjin University, Tianjin, 300072, P.R. China

*Corresponding Author: Ying-Jin Yuan, Email: yjyuan@tju.edu.cn, Tel: 86-22-27403888, Postal address: No. 92, Weijin Road, Nankai District, Tianjin, 300072, PR China

Co-authors:

Xiao-Jing Guo (Email: xjguo@tju.edu.cn)

Wen-Hai Xiao (Email: wenhai.xiao@tju.edu.cn)

Ying Wang (Email: [ying.wang@tju.edu.cn](mailto:ying.wang@tju.edu.cn))

Ming-Dong Yao (Email: [mingdong.yao@tju.edu.cn](mailto:mingdong.yao@tju.edu.cn))

Bo-Xuan Zeng (Email: [zengboxuan@126.com](mailto:zengboxuan@126.com))

Hong Liu (Email: season_l@sina.com)

Guang-Rong Zhao（Email:grzhao@tju.edu.cn）

**Table S1.** Oligonucleotides used in this study.

| **Oligos** | **Sequence (5’- 3’)** |
| --- | --- |
| **For construction of cassette** *ADE1*_L- T_IDI1_-*IDI1*-P_GAL10_-P_GAL1_-*tHMG1*-T_tHMG1_- *ADE1*-*ADE1* _R | |
| A-ADE1up-F | CGAATTCCTGCAGTTTAAACTACTCAGTATATTAAGTTTCGA |
| A-ADE1up-R | GTTGTTGTAATTGTCATAACTCGTGAATAAGCTTCGGGTG |
| A-tHMG1-F | CACCCGAAGCTTATTCACGAGTTATGACAATTACAACAAC |
| A-tHMG1-R | GGAGAAAAAACTATAATGGCTGCAGACCAATTGGTGAAAAC |
| A-pGAL1,10-F | GTTTTCACCAATTGGTCTGCAGCCATTATAGTTTTTTCTCC |
| A-pGAL1,10-R | TATTGTTGTCGGCAGTCATTTATATTGAATTTTCAAAAAT |
| A-IDI1-F | ATTTTTGAAAATTCAATATAAATGACTGCCGACAACAATA |
| A-IDI1-R | CTCGCAAGAGTCAGACTGACTTAAATAAAGAAAATAAAGTT |
| A-ADE1-F | AACTTTATTTTCTTTATTTAAGTCAGTCTGACTCTTGCGAG |
| A-ADE1-R | CCCCGGGCTGCAGTTTAAACTTAGTGAGACCATTTAGACC |
| **For construction of cassette** *URA3*_L-T_ERG13_-*ERG13*-P_GAL10_-P_GAL1_-*tHMG1*-T_tHMG1_-*URA3*-*URA3*_R | |
| U-URA3-up-F | CGAATTCCTGCAGTTTAAACTTGCTAAATTCGAGTGAAACAC |
| U-URA3-up-R | TTGTTGTAATTGTCATAACTATGGACCCTGAAACCACAGC |
| U-tHMG1-F | GCTGTGGTTTCAGGGTCCATAGTTATGACAATTACAACAA |
| U-tHMG1-R | CAAGGAGAAAAAACTATAATGGCTGCAGACCAATTGGTGAAAACT |
| U-pGAL1,10-F | AGTTTTCACCAATTGGTCTGCAGCCATTATAGTTTTTTCTCCTTG |
| U-pGAL1,10-R | AACAAAGTTTAGTTGAGAGTTTCATTTATATTGAATTTTCAAAAAT |
| U-ERG13-F | ATTTTTGAAAATTCAATATAAATGAAACTCTCAACTAAACTTTGTT |
| U-ERG13-R | GAATTGAATTGAAAAGCGTGGTTATATATATATCATTGTTAT |
| U-URA3-F | ATAACAATGATATATATATAACCACGCTTTTCAATTCAATTC |
| U-URA3-R | CCCCGGGCTGCAGTTTAAACTTAGTTTTGCTGGCCGCATC |
| **For construction of cassette** *TRP1*_L- T_ERG20_-*ERG20*-P_GAL10_-P_GAL1_-*tHMG1*-T_tHMG1_-*TRP1*-*TRP1*_R | |
| T-TRP1 up F | cgaattcctgcagtttaaacCTACTATTAGCTGAATTGCC |
| T-TRP1 up R | GTTGTTGTAATTGTCATAACTTGTCAGCTCTTTTAGATCGG |
| T-tHMG1 F | CCGATCTAAAAGAGCTGACAAGTTATGACAATTACAACAAC |
| T-tHMG1 R | CAAGGAGAAAAAACTATAATGGCTGCAGACCAATTGGTGAAAACT |
| T-pGAL1,10 F | AGTTTTCACCAATTGGTCTGCAGCCATTATAGTTTTTTCTCCTTG |
| T-pGAL1,10 R | GTCCAAGGATTTTTTTTAAACCATAAACGACATTACTAT |
| T-ERG20 F | AATTTTTGAAAATTCAATATAAATGGCTTCAGAAAAAGAAATTAGGA |
| T-ERG20 R | ATAGTAATGTCGTTTATGGTTTAAAAAAAATCCTTGGAC |
| T-TRP1 F | TCCTAATTTCTTTTTCTGAAGCCATTTATATTGAATTTTCAAAAATT |
| T-TRP1 R | ccccgggctgcagtttaaacCTATTTCTTAGCATTTTTGACG |
| **For construction of cassette** *LEU2*_F-*BieR-ERG19-*P_GAL1,10_*-ERG8- LEU2*_R | |
| L-Leu2up-F | acggtatcgataagcttgatatcgaattcctgcaggtttaaacTTTTCCAATAGGTGGTTAGCAA |
| L-Leu2up-R | GTTGTCTACCATGATCCAATATCGTCTTCCTTTCTCTTACCAAAGTA |
| BleR-F | TACTTTGGTAAGAGAAAGGAAGACGATATTGGATCATGGTAGACAACCC |
| BleR-R | TTACCACTTGAATGAGAAACGCTCCTAGTGGATCTGATATCAC |
| L-ERG19-F | atatcagatccactaggagcGTTTCTCATTCAAGTGGTAACTGC |
| L-ERG19-R | TAACGTCAAGGAGAAAAAACTATAATGACCGTTTACACAGCATCCGTTACC |
| L-pGAL1,10-F | GGTAACGGATGCTGTGTAAACGGTCATTATAGTTTTTTCTCCTTGACGTTA |
| L-pGAL1,10-R | CACTGAAGGCTCTCAACTCTGACATTTATATTGAATTTTCAAAAATT |
| L-ERG8-F | AATTTTTGAAAATTCAATATAAATGTCAGAGTTGAGAGCCTTCAGTG |
| L-ERG8-R | CAAATATCATAAAAAAAGAGAATCTTTTAGCTTGTACCCATTAAAAGAATTTTATCATGCCG |
| L-Leu2down-F | CTTTTAATGGGTACAAGCTAAAAGATTCTCTTTTTTTATGATATT |
| L-Leu2down-R | GGCCGCTCTAGAACTAGTGGATCCCCCGGGCTGCagtttaaacTTAACGTTTCTTTCGCCTACGTGGAAGGAGAATC |
| **For construction of cassette** *HIS3-ERG12-* P_GAL1,10_*-ERG10*- *HIS3*_R | |
| His3-F | gacggtatcgataagcttgatatcgaattcctgcagtttaaacATGACAGAGCAGAAAGCCCTAG |
| His3-R | ATGAAATTTAAGCAGACCATTATGATCCGTCGAGTTCAAGAG |
| H-ERG12-F | cttgaactcgacggatcataATGGTCTGCTTAAATTTCATTCTG |
| H-ERG12-R | TAACGTCAAGGAGAAAAAACTATAATGTCATTACCGTTCTTAACTTCTGCA |
| H-pGAL1,10-F | TGCAGAAGTTAAGAACGGTAATGACATTATAGTTTTTTCTCCTTGACGTTA |
| H-pGAL1,10-R | ATACAATGTAAACGTTCTGAGACATTTATATTGAATTTTCAAAAATT |
| H-ERG10-F | AATTTTTGAAAATTCAATATAAATGTCTCAGAACGTTTACATTGTAT |
| H-ERG10-R | CTCCATCTCTTTTATATTTTTTTTCAAAAGTAAGTCAAAAGGCAC |
| H-His3down-F | TGTGCCTTTTGACTTACTTTTGAAAAAAAATATAAAAGAGATGGAGGAACGG |
| H-His3down-R | GGCCGCTCTAGAACTAGTGGATCCCCCGGGCTGCAGTTTAAACCGATAGGGACGGAGTATTTTTG |
| **For construction of cassette** *GAL1,7, 10*_L *-HphR- GAL1,7, 10*_R | |
| GAL7,10,1-HphF | caggcagttaatagaaaaaatatgatatgaatgaatattccactttctttTAGGTCTAGAGATCTGTTTAGCTTGC |
| HphR-GAL1,7, 10-R | TGAGTAGAAAAAAATGAGAAGTTGTTCTGAACAAAGTAAAAAAAAGAAGTATTAAGGGTTCTCGAGAGCTCG |
| T_FBA1_-F | aaaactgcaggcggccgcGTTAATTCAAATTAATTGATATAGTTTTTTAATG |
| T_FBA1_-R | AAGGATAGTAAGCTGGCAAAAAAGATGAGCTAGGCTTTTGTAAAAATATC |
| P_GAL7_-F | caaaagcctagctcatctttTTTGCCAGCTTACTATCCTTCTTG |
| P_GAL7_-R | CGATTTCAATTCAATTCAATTTTAGGAGACCGGTCTCCCAT |
| T_PGK1_-F | tgggagaccggtctcctaaaATTGAATTGAATTGAAATCGATAG |
| T_PGK1_-R | CGCGGATCCGCGGCCGCAACGAACGCAGAATTTTCGAG |
| **For construction of cassette** T_TDH2_-P_GAL1_-T_CYC1_ | |
| T_TDH2_-P_GAL1__F | AAAACTGCAGGCGGCCGCATTTAACTCCTTAAGTTACTTTAATGATTTAG |
| T_TDH2_-P_GAL1__R | TTTAGGAGACCGGTCTCCC |
| T_CYC1__F | GGGAGACCGGTCTCCTAAAC |
| T_CYC1__R | CGCGGATCCGCGGCCGCAAAGCCTTCGAGCGTCCC |
| **For construction of cassette** T_TDH2_-P_GAL7_-T_CYC1_ | |
| T_TDH2__F | AAAACTGCAGGCGGCCGCATTTAACTCCTTAAGTTACTTTAATGATTTAG |
| T_TDH2__R | AAGGATAGTAAGCTGGCAAAGCGAAAAGCCAATTAGTGTG |
| P_GAL7__F | CACACTAATTGGCTTTTCGCTTTGCCAGCTTACTATCCTTC |
| P_GAL7__R | TTTAGGAGACCGGTCTCCCATTTTTTGAGGGAATATTCAACTGTT |
| T_CYC1__F | AGTTGAATATTCCCTCAAAAAATGGGAGACCGGTCTCC |
| T_CYC1__R | CGCGGATCCGCGGCCGCAAAGCCTTCGAGCGTCCC |
| **For construction of cassette** *GAL80*_L-*LEU2*-T_FBA1_ | |
| GAL80_F_1_ | AAAACTGCAGGCGGCCGCTCTGCCATGGCAAAGAATG |
| GAL80_F_2_ | CTCTAAAAAAAAAAAAATACGACGGGAGTGGAAAGAACG |
| LEU2_F | CCGTTCTTTCCACTCCCGTCGTATTTTTTTTTTTTTAGAGAAAATCC |
| LEU2_R | ATCAATTAATTTGAATTAACTTATTTTTTTCCTCAACATAACGAG |
| T_FBA1_-F | TATGTTGAGGAAAAAAATAAGTTAATTCAAATTAATTGATATAGTTTTTTAATG |
| T_FBA1_-R | CGCGGATCCGCGGCCGCAAAGATGAGCTAGGCTTTTGTAAAAATATC |
| **For construction of cassette** T_PGK1-_*GAL80*_R | |
| T_PGK1_-F | AAAACTGCAGGCGGCCGCATTGAATTGAATTGAAATCGATAG |
| T_PGK1_-R | AAGCACAGGGCAAGATGCTTAACGAACGCAGAATTTTCGAG |
| GAL80_R_1_ | TCGAAAATTCTGCGTTCGTTAAGCATCTTGCCCTGTGCT |
| GAL80_R_2_ | CGCGGATCCGCGGCCGCCCGTTTCCTTAATTACATAAAGC |
| **For construction of cassette** *GAL7,10,1*_L-*URA3*-T_TDH2_ | |
| GAL7,10,1_F_1_ | AAGGAAAAAAGCGGCCGCACAATTCTGAAAACCAAAGACTG |
| GAL7,10,1_F_2_ | GGAATTGCCATGAAGCCGAAAAAGAAAGTGGAATATTCATTCATATC |
| URA3-F | ATGAATATTCCACTTTCTTTTTCGGCTTCATGGCAATTC |
| URA3-R | AGTAACTTAAGGAGTTAAATGGTAACGCCAGGGTTTTCC |
| T_TDH2_-F | GGGAAAACCCTGGCGTTACCATTTAACTCCTTAAGTTACTTTAATGATTTAG |
| T_TDH2_-R | TTTTCCTTTTGCGGCCGCGCGAAAAGCCAATTAGTGTG |
| **For construction of cassette** T_CYC1-_*GAL7,10,1*_R | |
| T_CYC1_-F | AAAACTGCAGGCGGCCGCCATGTAATTAGTTATGTCACGCTTAC |
| T_CYC1_-R | ACAAAGTAAAAAAAAGAAGTAAAGCCTTCGAGCGTCCC |
| GAL7,10,1_R_1_ | TTGGGACGCTCGAAGGCTTTACTTCTTTTTTTTACTTTGTTCAGAAC |
| GAL7,10,1_R_2_ | CGCGGATCCGCGGCCGCAGAACAGAGATTTAATGGAAAATG |
| **For construction of cassette** *ERG6*_L-*LEU2*-T_TDH2_ | |
| ERG6_F_1_ | AAAACTGCAGGCGGCCGCTAATCGGTAAACGGAAGACTATTAAG |
| ERG6_F_2_ | TATGTTGAGGAAAAAAATAACTTATGCTGCCTACTATATTATTATTTTATTC |
| LEU2-F | AATATAGTAGGCAGCATAAGTTATTTTTTTCCTCAACATAACGAG |
| LEU2-R | AGTAACTTAAGGAGTTAAATGTATTTTTTTTTTTTTAGAGAAAATCC |
| T_TDH2_-F | CTCTAAAAAAAAAAAAATACATTTAACTCCTTAAGTTACTTTAATGATTTAG |
| T_TDH2_-R | CGCGGATCCGCGGCCGCGCGAAAAGCCAATTAGTGTG |
| **For construction of cassette** T_CYC1_-*ERG6*_R | |
| T_CYC1_-F | AAAAGAATTCGCGGCCGCCATGTAATTAGTTATGTCACGCTTAC |
| T_CYC1_-R | ATCTTATTGATCTAGTGAATAAAGCCTTCGAGCGTCCC |
| ERG6_R_1_ | TTGGGACGCTCGAAGGCTTTATTCACTAGATCAATAAGATTCAAATAAAG |
| ERG6_R_2_ | CGCGGATCCGCGGCCGCGTAATTTTATATAACTTCTTTTTCGTTAGC |
| **For construction of cassette** *ERG6*_L-*LEU2*-*ERG6*_R | |
| ERG6_F_1_ | AAAACTGCAGGCGGCCGCTAATCGGTAAACGGAAGACTATTAAG |
| ERG6_F_2_ | TATGTTGAGGAAAAAAATAACTTATGCTGCCTACTATATTATTATTTTATTC |
| LEU2-F | AATATAGTAGGCAGCATAAGTTATTTTTTTCCTCAACATAACGAG |
| LEU2-R | ATCTTATTGATCTAGTGAATGTATTTTTTTTTTTTTAGAGAAAATCCTC |
| ERG6_R_1_ | CTCTAAAAAAAAAAAAATACATTCACTAGATCAATAAGATTCAAATAAAG |
| ERG6_R_2_ | CGCGGATCCGCGGCCGCGTAATTTTATATAACTTCTTTTTCGTTAGC |
| **For construction of cassette** *FLD1*_L-*URA3*-*FLD1*_R | |
| FLD1_F_1_ | AAGGAAAAAAGCGGCCGCAAGAAAAGAAACTTTAATTCTTCTTATTC |
| FLD1_F_2_ | TGAATTGAATTGAAAAGCTTCCTTCCTATTCACTTTATCTTATTTTC |
| URA3-F | AGATAAAGTGAATAGGAAGGAAGCTTTTCAATTCAATTCATCAT |
| URA3-R | TAGCGAGAAGTACAATTCTACCCGGGTAATAACTGATATAATTAAAT |
| FLD1_R_1_ | TATATCAGTTATTACCCGGGTAGAATTGTACTTCTCGCTATATAATTTTAAAAC |
| FLD1_R_2_ | CGCGGATCCCTTATTTTCGTTTCAATTCTTAGTTTG |
| **For construction of cassette** *NEM1*_L-*URA3*-*NEM1*_R | |
| NEM1_F_1_ | AAGGAAAAAAGCGGCCGCTTGTGCATACCCACGATCC |
| NEM1_F_2_ | TGAATTGAATTGAAAAGCTTCAATTGTTATTGTGTCCTTCCA |
| URA3-F | GAAGGACACAATAACAATTGAAGCTTTTCAATTCAATTCATCAT |
| URA3-R | GACATTGTTTCATTAATTGACCCGGGTAATAACTGATATAATTAAATTG |
| NEM1_R_1_ | TATATCAGTTATTACCCGGGTCAATTAATGAAACAATGTCCAG |
| NEM1_R_2_ | CGCGGATCCCTAAGTGGTTCAAAGATACACCG |
| **For construction of cassette** *FLD1_*L*-KanMX*-*FLD1*_R | |
| *FLD*1_L-F | AAGGAAAAAAGCGGCCGCAAGAAAAGAAACTTTAATTCTTCTTATTC |
| *FLD*1_L-R | TAAACAGATCTCTAGACCTACCTTCCTATTCACTTTATCTTATTTTC |
| G418-F | AGATAAAGTGAATAGGAAGGTAGGTCTAGAGATCTGTTTAGCTTGC |
| G418-R | TAGCGAGAAGTACAATTCTAATTAAGGGTTCTCGAGAGCTCG |
| *FLD*1_R-F | AGCTCTCGAGAACCCTTAATTAGAATTGTACTTCTCGCTATATAATTTTAAAAC |
| *FLD*1_R-R | CGCGGATCCCTTATTTTCGTTTCAATTCTTAGTTTG |
| **For adding polyhistidine Tag to DHCR24s from diversity sources** | |
| DHCR24(*Cg*)_his-F | aaaggtctccaATGGCTGTTACTCAAGATAATTTG |
| DHCR24(*Cg*)_his-R | acaggtctcgtTTAGTGGTGGTGGTGGTGGTGATAGATACCTGGAATGAACTTCCA |
| DHCR24(*Tg*)_his-F | aaaggtctccaATGACTACAAAATCCAGAAGAGC |
| DHCR24(*Tg*)_his-R | acaggtctcgtTTAGTGGTGGTGGTGGTGGTGATAGATACCAGGTATAAACTTGTATGG |
| DHCR24(*Mm*)_his-F | aaaggtctccaATGGAACCAGCTGTTTCTTTAG |
| DHCR24(*Mm*)_his-R | acaggtctcgtTTAGTGGTGGTGGTGGTGGTGGTGTCTAGCGGCCTTGCAG |
| DHCR24(*At*)_his-F | aaaggtctccaATGAGTGATTTGCAAACTCCATTAG |
| DHCR24(*At*)_his-R | acaggtctcgtTTAGTGGTGGTGGTGGTGGTGATCAGCTTCGGCATATGCTG |
| DHCR24(*Dr*)_his-F | aaaggtctccaATGGACCCATTGTTATACTTGG |
| DHCR24(*Dr*)_his-R | acaggtctcgtTTAGTGGTGGTGGTGGTGGTGGTGTCTGGCTGACTTGCAAAT |
| DHCR24(*Gh*)_his-F | aaaggtctccaATGTCTGACTTGCAAGCACC |
| DHCR24(*Gh*)_his-R | acaggtctcgtTTAGTGGTGGTGGTGGTGGTGATCAGCTTCGGCATATGCAG |
| DHCR24(*Ec*)_his-F | aaaggtctccaATGGAACCAGCTGTCTCTTTAG |
| DHCR24(*Ec*)_his-R | acaggtctcgtTTAGTGGTGGTGGTGGTGGTGGTGTCTAGCGGCCTTGCAG |
| DHCR24(*Gg*)_his-F | aaaggtctccaATGAGTGCCGTTTGGTCTTTAG |
| DHCR24(*Gg*)_his-R | acaggtctcgtTTAGTGGTGGTGGTGGTGGTGATGTCTAGCGGCTTTGCAAATC |
| DHCR24(*Xt*)_his-F | aaaggtctccaATGGAATTGTTATTGTACGTTGG |
| DHCR24(*Xt*)_his-R | acaggtctcgtTTAGTGGTGGTGGTGGTGGTGATGTCTAGCGGCCTTGCATATC |
| DHCR24(*Bt*)_his-F | aaaggtctccaATGGAACCAGCTGTTTCATTG |
| DHCR24(*Bt*)_his-R | acaggtctcgtTTAGTGGTGGTGGTGGTGGTGGTGTCTAGCGGCCTTGCAG |
| DHCR24(*Hs*)_his-F | aaaggtctccaATGGAGCCCGCTGTTTCTC |
| DHCR24(*Hs*)_his-R | acaggtctcgtTTAGTGGTGGTGGTGGTGGTGGTGTCGAGCAGCCTTACAGATC |

**Table S2.** The Codon-optimized sequences of DHCR24s involved in this study.

| **Protein** | **Encoding sequences** |
| --- | --- |
| DHCR24 from *Homo sapiens* (*HS_*DHCR24) | atggagcccgctgtttctctggctgtttgtgctctgctgttcctgctgtgggttcgactgaagggtctggagttcgttctgatccaccagcgatgggttttcgtttgtctgttcctgctgcccctgtctctgatcttcgacatctactactacgttcgagcttgggttgttttcaagctgtcttctgctccccgactgcacgagcagcgagttcgagacatccagaagcaggttcgagagtggaaggagcagggttctaagaccttcatgtgtaccggtcgacccggttggctgaccgtttctctgcgagttggtaagtacaagaagacccacaagaacatcatgatcaacctgatggacatcctggaggttgacaccaagaagcagatcgttcgagttgagcccctggttaccatgggtcaggttaccgctctgctgacctctatcggttggaccctgcccgttctgcccgagctggacgacctgaccgttggtggtctgatcatgggtaccggtatcgagtcttcttctcacaagtacggtctgttccagcacatctgtaccgcttacgagctggttctggctgacggttctttcgttcgatgtaccccctctgagaactctgacctgttctacgctgttccctggtcttgtggtaccctgggtttcctggttgctgctgagatccgaatcatccccgctaagaagtacgttaagctgcgattcgagcccgttcgaggtctggaggctatctgtgctaagttcacccacgagtctcagcgacaggagaaccacttcgttgagggtctgctgtactctctggacgaggctgttatcatgaccggtgttatgaccgacgaggctgagccctctaagctgaactctatcggtaactactacaagccctggttcttcaagcacgttgagaactacctgaagaccaaccgagagggtctggagtacatccccctgcgacactactaccaccgacacacccgatctatcttctgggagctgcaggacatcatccccttcggtaacaaccccatcttccgatacctgttcggttggatggttccccccaagatctctctgctgaagctgacccagggtgaaaccctgcgaaagctgtacgagcagcaccacgttgttcaggacatgctggttcccatgaagtgtctgcagcaggctctgcacaccttccagaacgacatccacgtttaccccatctggctgtgtcccttcatcctgccctctcagcccggtctggttcaccccaagggtaacgaggctgagctgtacatcgacatcggtgcttacggtgagccccgagttaagcacttcgaggctcgatcttgtatgcgacagctggagaagttcgttcgatctgttcacggtttccagatgctgtacgctgactgttacatgaaccgagaggagttctgggagatgttcgacggttctctgtaccacaagctgcgagagaagctgggttgtcaggacgctttccccgaggtttacgacaagatctgtaaggctgctcgacactaa |
| DHCR24 from *Mus musculus* (*Mm_*DHCR24) | atggaaccagctgtttctttagcagtctgtgccttgttatttttgttgtgggtcagagtaaagggtttggaattcgttttgatccatcaaagatgggttttcgtctgcttgttcttgttaccattgtctttgatattcgatatctattactatgttagagcatgggttgtcttcaaattatcttcagcccctagattgcatgaacaaagagttagagatattcaaaagcaagtcagagaatggaaagaacaaggtagtaagacttttatgtgtacaggtagaccaggttggttgacagtatctttgagagttggtaaatacaaaaagacccataagaacatcatgatcaacttgatggatatcttggaagttgacactaaaaagcaaatcgtaagagttgaacctttagtctcaatgggtcaagtaaccgcattgttaaattccattggttggactttgccagtcttacctgaattggatgacttaactgtaggtggtttaatcatgggtacaggtattgaatccagttctcataagtacggtttgttccaacacatctgtactgcttacgaattgatcttggcagatggttcattcgttagatgcacaccatcagaaaactccgacttgttctacgcagtaccttggtcctgtggtacattgggtttcttggttgctgctgaaataagaatcatcccagctaaaaagtacgtaaagttgagattcgaacctgttagaggtttggaagcaatatgtgaaaagtttaccagagaatcacaaagattggaaaaccatttcgttgaaggtttgttgtactccttggatgaagccgttatcatgaccggtgtcatgactgatgacgtagaaccatcaaagttgaacagtatcggttcttactacaagccttggtttttcaagcatgttgaaaactacttgaagaccaacagagaaggtttagaatacatcccattgagacactactaccatagacacactagatcaatcttttgggaattacaagatatcataccattcggtaacaaccctatttttagatacttattcggttggatggttccacctaaaatatccttgttaaagttgacacaaggtgaaaccttgagaaaattgtacgaacaacatcacgtagttcaagatatgttagttcctatgaagtgtatgagtcaagctttgcatacattccaaaatgatatacacgtctatccaatctggttatgcccttttatattgccatctcaacctggtttagttcatccaaaaggtgacgaagccgaattgtacgttgacatcggtgcttatggtgaacctagagtcaaacatttcgaagccagaagttgtatgagacaattggaaaagttcgtcagatctgtacacggtttccaaatgttgtacgctgattgctacatgaacagagaagaattctgggaaatgttcgacggttctttgtaccataagttgagaaagcaattgggttgtcaagatgcattcccagaagtttacgacaagatctgcaaggccgctagacactaa |
| DHCR24 from *Danio rerio* (*Dr_*DHCR24) | atggacccattgttatacttgggtggtttagctgttttgtttttaatctggatcaaagtaaagggtttagaatacgttataatacatcaaagatggatttttgtttgtttattcttgttaccattgtcagttgtctttgatgtctattaccatttgagagcatggatcattttcaaaatgtgctccgcccctaagcaacacgatcaaagagtcagagacattcaaagacaagtaagagaatggagaaaagatggtggtaaaaagtacatgtgtactggtagaccaggttggttgacagtctcattaagagttggtaaatacaaaaagactcataagaacatcatgatcaacatgatggatatcttggaagtagacacaaagagaaaggtagttagagttgaacctttggccaatatgggtcaagtcacagctttgttaaactccataggttggaccttgccagttttacctgaattggatgacttaaccgttggtggtttagtcatgggtactggtatcgaatcttcatcccatatctatggtttgtttcaacacatttgtgtagctttcgaattggttttagcagatggttctttagttagatgcaccgaaaaagaaaattcagacttgttttacgccgtcccatggtcctgtggtacattgggtttcttagtagctgctgaaatcagaatcatcccagctcaaaaatgggttaagttacattatgaacctgtcagaggtttggatgcaatctgcaaaaagtttgcagaagaaagtgccaataaggaaaaccaattcgttgaaggtttacaatactctagagacgaagctgtcatcatgaccggtgtaatgactgatcatgcagaaccagacaagactaactgtatcggttactactacaagccttggtttttcagacatgttgaatcatttttgaagcaaaacagagttgcagtcgaatacataccattgagacactactaccatagacacacaagatctatattttgggaattacaagatatcatcccattcggtaacaaccctttgtttagatacgttttcggttggatggtcccacctaagatctctttgttgaagttgacacaaggtgaaaccatcagaaaattgtatgaacaacatcacgtcgtacaagatatgttggttcctatgaaggacataaaggccgctatccaaagattccatgaagatatccacgtttacccattgtggttatgccctttcttgttaccaaatcaacctggtatggttcatccaaaaggtgacgaagacgaattgtacgtagatattggtgcctatggtgaacctaaagttaagcattttgaagctactagttctacaagacaattggaaaagtttgtaagagatgttcacggtttccaaatgttgtacgctgacgtttacatggaaagaaaggaattctgggaaatgttcgatggtactttgtaccataagttgagagaagaattgggttgtaaggatgcatttccagaagttttcgacaaaatttgcaagtcagccagacactaa |
| DHCR24 *from Equus caballus* (*Ec_*DHCR24) | atggaaccagctgtctctttagcagtatgtgccttgttatttttgttgtggattagagtcaagggtttagaattcgtaatcatccatcaaagatgggtcttcgtatgcttgttcttgttaccattgtcattgatcttcgatatctattactatgttagagcatgggttgtcttcaaattaaattccgcccctagattgcatgaacaaagagttagaaacattcaaaagcaagtcagagaatggaaagaacaaggtagtaagacttttatgtgtacaggtagaccaggttggttgacagtttctttgagagtcggtaaatacaaaaagacccataagaacatcatgatcaacttgatggatatcttggaagttgacactaagaaacaaatcgttagagtcgaacctttagtaactatgggtcaagttacagccttgttaaattcaattggttggaccttgccagttttacctgaattggatgacttaaccgtcggtggtttaattatgggtactggtatagaatcttcatcccataagtacggtttgttccaacacatctgtactgcctatgaattggtattagctgatggtagttttgttagatgcacaccaagtgaaaattctgacttgttctacgcagtcccttggtcttgtggtactttgggtttcttggtagctgctgaaatcagaatcatcccagctaagaaatacgtcaagttgagattcgaacctgtaagaggtttggaagccatctgtgataagtttgctttagaagcacaaagaccagaaaacgacttcgttgaaggtttgttatacagtttggaagaagctgtcatcatgaccggttctatgactgataaagcagaaccatcaaagttgaactccatcggtaactactacaagccttggtttttcaagcatgttgaaaactacttgaagaccaaccaagaaggtttagaatacatccctttgagacactactaccatagacacactagatcaatattttgggaattgcaagatatcatcccattcggtaacaaccctgtattcagatactttttcggttggatggttccacctaagatctccttgttgaagttgacacaaggtgaaaccttgagaaaattgtacgaacaacatcacgtagttcaagatatgttggttccaatgaagtgtttgcaacaagctttgcatactttccaaaacgatatccacgtttacccaatatggttgtgcccttttattttgccatcacaacctggtttagttcatccaaaaggtgacgaaacagaattgtacatcgacattggtgcctatggtgaacctagagttaaacatttcgaagctagatcatgtatgagacaattggaaaagttcgtaagatccgttcacggtttccaaatgttgtacgctgattgctatatgaacagagaagaattttgggaaatgttcgacggttctttgtaccataagttgagaaagcaattgggttgtgaagatgcattcccagaagtttacgacaagatctgcaaggccgctagacactaa |
| DHCR24 from *Gallus gallus* (*Gg_*DHCR24) | atgagtgccgtttggtctttaggtgctggtttgttgttgttgttgttgtgggttagacatagaggtttagaagctgttttggtccatcacagatggatcttcgtttgtttctttttgatgccattgtctatcttgttcgatgtatactaccaattaagagcatgggccgttagaagaatgcattcagcacctagattgcacggtcaaagagtcagacatattcaagaacaagtaagagaatggaaagaagaaggtggtagaagatatatgtgcacaggtagaccaggttggttaaccgtttccttgagagtcggtaaatacaaaaagactcataagaacatcatgataaacttaatggatgtattggaagttgactctgaaagacaagttgtcagagtagaaccattggttaccatgggtcaattaactgcttatttgaatcctatgggttggacaattccagtagttcctgaattagatgacttgactgttggtggtttaataatgggtacaggtatcgaatcttcatcccacatctatggtttgtttcaacatacctgtatggcatacgaattggttttagccgatggttcattagtcagatgctccccaacagaaaacagtgacttgttttatgccgttccttggtcttgtggtaccttaggtttcttggtcgctgctgaaattaaaatgatcccagctaaaaagtacatcagattgcattacgaacctgttagaggtttgagatcaatctgcgaaaagtttactgaagaatctaaaaataaggaaaactcattcgtcgaaggtttagtatactccttggaagaagctgtaattatgactggtgttttaacagatgaagcagaacctagtaagattaatagaatcggtaactactacaagccttggtttttcaagcacgttgaaaagtatttgaaggccaataagactggtatcgaatacattccatccagacattactaccatagacacacaagaagtattttctgggaattacaagatatcatcccattcggtaacaaccctgtctttagatatttgttcggttggatggtaccacctaagatctctttgttgaagttgacccaaggtgaagcaattagaaaattgtacgaacaacatcacgtcgtacaagatatgttagttcctatgaagtcattggaaaaatccatccaaacttttcacgttgacttaaacgtctatccattgtggttatgtcctttcttgttaccaaataaccctggtatggttcatccaaagggtgacgaaaccgaattgtatgttgacataggtgcttacggtgaacctaaaactaagcaatttgaagctagagcatctatgagacaaatggaaaaatttgtcagatcagtacatggtttccaaatgttgtacgcagattgttatatgactagagaagaattttgggatatgttcgacggtagtttataccactctttgagagaacaaatgaactgtaaggatgcctttccagaagtttacgacaagatttgcaaagccgctagacattaa |
| DHCR24 from *Xenopus tropicalis* (*Xt_*DHCR24) | atggaattgttattgtacgttggtggtttattgattttcttgttgttgtggatgagagctaaaggtttcgaatatgttatcgtccatcacagatggatcttcgtttgtttattcttgttgccattgtcagtcatattcgatgtatactactacgcaagagcctggttggttttcaagatgtgctccgctcctaagcaacatgatagaagagtcaaggatattcaagaccaagtaagacaatggaaggcagaaggtggtaaaaattttatgtgtactggtagaccaggttggttgacagttagtttgagagtcggtaaatataaaaacacccacaagaacatcatgataaatttgatggatatcttggaagtagacactaaaagacaagttgtcagagtagaacctttggttaacatgggtcaagttacagctttgttgaactctataggttggaccttaccagtagttcctgaattggatgacttaaccgttggtggtttgattatgggtactggtatagaatcttcatcccataacttcggtttattccaacacatatgtttggcttacgaattggttttagcagatggttcattagtcagatgcactccaacagaaaatagtgacttgttttatgccgtcccttggtcttgtggtacattgggtttcttagtagctgctgaaattaaaatcgtaccagctaagaaatacgttaagttgcattacacccctgttaagggtttggaaaagatttgcgaaaaattttctagagaatctaaaaataaggaaaactacttcgtcgaaggtttagtatattctgccgatgaagctgttattatgaccggtgtcttgactgacgaagctcaagcaggtcaagttaacagaataggtcaatactggaagccatggtttttcagacatgtcgaaagttatttgaaaaataacagagatggtactgaatacatccctttgagacactactaccatagacacacaaagtctatcttttgggaattacaagatatcatcccattcggtaaccatcctgtattcagatactttttcggttggatggttccacctaagatctcattgttgaagttgacacaaggtgaaaccatcagaaagttgtacgaacaacatcacgtcgtacaagatatgttagtcccaatgaagtgtttgcaaaaagcaataactgcctttcattcagaaatctccgtttatccattgtggttatgccctttcattttaccatcccatcctggtatgatacacccaaagggtaacgaagccgaattgtacgttgatattggtgcttatggtgaacctaaaacaaagcattttgacgctaagggttcaatgagaagattggaaaagttcgtaagagatgttcacggtttccaaatgttgtacgcagactgttatatgtccagagaagaattttgggatatgttcgacggtgcattgtaccaaaagttgcgtggtaaattgaactgtaacaacgccttcccagaagtttacgataagatatgcaaggccgctagacattaa |
| DHCR24 from  *Bos taurus* (*Bt_*DHCR24) | atggaaccagctgtttcattggcagtctgtgccttgttatttttgttgtgggtcagagtaaagggtttggaattcgttttgatccatcaaagatgggttttcgtctgcttattcttgttaccattgtccttgatattcgatatctattactacgttagagcatgggttgtcttcaaattatcttcagcccctagattgcatgaacaaagagtaagagatatccaaaagcaagttagagaatggaaagaacaaggttcaaagacttttatgtgtacaggtagaccaggttggttgacagtatccttgagagttggtaaatacaaaaagacccataagaacatcatgatcaacttgatggatatcttggaagttgacactaaaaagcaaatcgtaagagttgaacctttggtcacaatgggtcaagtaaccgctttgttaacttcaattggttggactttgccagttttacctgaattggatgacttaaccgtcggtggtttaatcatgggtactggtattgaatccagttctcatagatacggtttgtttcaacacatatgtaccgcttatgaattggtcttagcagatggttcttttgtaagatgcactccaatggaaaatagtgacttattctacgcagtcccttggtcttgtggtactttgggtttcttggtagctgctgaaatcagaattataccagccaaaaagtacataaagttgagattcgaacctgttagaggtttggaagctatctgtgataagtttacacatgaaagtcaacaaccagaaaaccacttcgttgaaggtttgttgtactctttgcatgaagctgttatcatgacaggtgtcatgaccgatgaagcagaaccatcaaagttgaactccattggtaactactacaaaccttggtttttcaagcatgttgaaaactacttaaagacaaacagagaaggtttagaatacatcccattgagacactactaccatagacacaccagaagtatcttttgggaattgcaagatatcattccattcggtaacaaccctatttttagatacttattcggttggatggttccacctaagatctctttgttgaagttgactcaaggtgaaacattgagaaaattgtacgaacaacatcacgtagttcaagatatgttagttccaatgaagtgtttgcctcaagctttacatacattccacaatgacatacatgtttatccaatctggttatgcccttttatattgccatctcaacctggtttagtccacccaaaaggtgacgaagccgaattgtacgtagacatcggtgcttatggtgaacctagagttaaacatttcgaagcaagatcatgtatgagacaattggaaaagttcgtcagatccgtacacggtttccaaatgttgtacgccgattgctatatggacagagaagaattttgggaaatgttcgatggttcattgtaccatagattgagaaagcaattgggttgtcaagatgcattcccagaagtttacgacaagatctgcaaggccgctagacactaa |
| DHCR24 from  *Arabidopsis thaliana*  (*At_*DHCR24) | atgagtgatttgcaaactccattagttagacctaagagaaagaaaacttgggtagactactttgttaagttcagatggatcatcgtcatctttattgtattgccattctctgctaccttctatttcttgatatatttgggtgacatgtggtccgagagtaagtctttcgaaaagagacaaaaggaacatgacgaaaacgtcaaaaaggtaatcaagagattgaaaggtagagatgcctcaaaggacggtttagtttgtactgcaagaaaaccttggattgccgtcggtatgagaaacgtagattataaaagagctagacactttgaagttgacttgggtgaattcagaaacatcttggaaatcaacaaggaaaagatgaccgcaagagttgaaccattagtcaatatgggtcaaatttcaagagccactgttcctatgaacttgtccttagcagttgtcgccgaattggatgacttaacagtcggtggtttgataaatggttatggtatcgaaggttcttcacatatctacggtttatttgcagatacagtagaagcctacgaaattgttttggctggtggtgaattagttagagcaaccagagataacgaatatagtgacttgtattacgcaataccatggtcacaaggtactttaggtttgttagttgctgctgaaataagattgataaaggtcaaggaatacatgagattgacatacatccctgttaagggtgacttgcaagccttagctcaaggttatatagactcatttgctccaaaggatggtgacaagtccaaaatccctgatttcgtagagggtatggtttataatccaaccgaaggtgttatgatggtcggtacttacgcttcaaaggaagaagctaaaaagaaaggcaacaaaattaataacgttggttggtggtttaagccatggttctatcaacatgctcaaacagcattgaagaaaggtcaattcgttgaatacatccctacaagagaatactaccatagacacaccagatgtttgtactgggagggtaaattgatcttaccattcggtgaccaattttggttcagatatttgttaggttggttgatgccacctaaggtttccttgttaaaagccactcaaggtgaagctatcagaaactactaccatgatatgcacgttatccaagacatgttggttccattatacaaagtcggtgacgcattagaatgggtccatagagaaatggaagtatatccaatttggttgtgtcctcacaagttgtttaaacaacctatcaaaggtcaaatctatccagaacctggtttcgaatacgaaaatagacaaggtgacactgaagacgcccaaatgtacacagatgtaggtgtttattacgctccaggttgcgtattgagaggtgaagaatttgatggttctgaagctgttagaagaatggaaaagtggttgatagaaaatcatggtttccaacctcaatatgcagttagtgaattggatgaaaagtctttttggagaatgttcaacggtgaattgtacgaagaatgcagaaagaaatacagagctataggtacctttatgtcagtctactacaagtccaagaaaggtagaaagactgaaaaagaagtaagagaagccgaacaagctcacttggaaacagcatatgccgaagctgattaa |
| DHCR24 from  *Gossypium hirsutum*  (*Gh_*DHCR24) | atgtctgacttgcaagcaccattaagacctaagagaaagaaaggtttggtagatttcttggttcaattcagatggatcttcgtcatatttttcgtattgccattctcaactttgtactactttttgatatatttgggtgacgttagatccgaaatgaagagttacaagcaaagacaaaaggaacatgatgaaaacgttttgaaggttgtcaagagattgaagcaaagaaacccaaagaaagatggtttggtctgtacagctagaaagccttggatagcagtcggtatgagaaatgtagactataaaagagctagacactacgaagttgatttgtccgcttttagaaacatcttggaaatagataagcaaagaatgattgctagagttgaaccattggtcaatatgggtcaaataaccagagtcactgtacctatgaacttgtccttagcagtagttgccgaattggatgacttaacagttggtggtttaatcaatggttatggtattgaaggttcttcacatatctacggtttgtttagtgacacagtcgtagcctatgaaattgttttagctgatggtagagttgtcagagcaaccaaggacaacgaatactctgatttgttttatgccatcccatggtcacaaggtactttgggtttcttggttgctgctgaaattaaattgatcccagtcaaggaatacatgagattaacttacacacctgtagttggtaatttgcaagacttagcacaaggttatatggattcatttgccccaagagatggtgaccaagataacccagaaaaagtacctgatttcgttgagggtatggtctattctcctacagaaggtgtttttatgaccggtagatacgcatcaaaggaagaagccaaaaagaaaggtaataagattaataacgttggttggtggttcaaaccatggttttatcaacatgctcaaacagcattgaagaaaggcgaattcgttgaatacatacctaccagagaatactaccatagacacactagatgtttgtactgggagggtaaattgatcttaccatttggtgaccaatggtggtttagattcttgttaggttggttgatgccacctaaagtttccttgttaaaggcaacccaaggtgaaagtatcagaaactactaccatgaaatgcacgtaatccaagacatgttggttcctttatacaaagtcggtgacgctttggaatgggttcatcacgaaatggaaatctatccaatatggttatgccctcatagattgttcaaattgccagtaaagactatggtttacccagaacctggttttgaacaacacagaagacaaggtgacactccttatgcccaaatgttcacagatgttggtgtctattacgctccaggtcctgtattgagaggtgaagtttttgatggtgccgaagctgtcagaaagttggaacaatggttaattaagaatcattccttccaaccacaatacgctgttagtgaattgaacgaaaaagatttctggagaatgtttgacgcagatttgtatgaacacgttagaagaaagtacggtgctgtcggtacttttatgtctgtatactacaagtcaaagaaaggtagaaagaccgaaaaggaagttcaagaagccgaacaagctcatttggaaactgcatatgccgaagctgattaa |
| DHCR24 from  *Cryptococcus gattii* (*Cg_*DHCR24) | atggctgttactcaagataatttgagacaaagaaaagctgcaaacttacaatctgatgacatagcaaatggtaactcaacagccttgttaaaaatcaatgctgttccaaccgaacatggtcaagaaagagataaggaattggacgaacaccaagaatatgaatttggtggtccaataggtgtattggctatgatgttaggtttccctgttttgatgtactacttatggatctgtttgtggtactaccaaggttcctttgtatatccaactagtgttgatgacattagaccatttttccatagaatgtgggaacacatctatgatggtgcatacccaacaaaatttgccttcattacttattggggtttaacagctatacaattggtctttgccgctgtaatgccaggcatgtaccaaaatggtttaccagttccttccttgaactacaagacattgccttacaagtgcaacgcattgtacagttggtactctactttgatcttagttggtgtcttgcataaaacaggtatctatagattgccatggatcatcgaaaacttcggtcacataatgaccgtttcaatcattacttcttactcagtctccataatcattgatgtttttgctagagtcttcaagtatggtggtggtcctttgagaatgtccggtaatatcttttacgatcatttcatgggtgtcagtttaaacccaagattgggtatcgtagacttaaagatgttcgcagaagtaagagttccttgggttttgttatttttgttcgcattatcagccactgttaagcaatatgaagaagcaggtagagtcacctacaatatgatacatttcttgttagccactggtttgtatatcaacgcatgtgccaaagctgaacaaatgattccacaaacctgggatatgtttcacgaaaagttcggttggatgttaattttctggaatatgtctggtgtcccaatgacatatgtataccctgctatctatatgtcaagagcacctattgaatcatacgaattctccagattgggtagttttgccttattctctacattgatgttgtgttactacatcttcgactgctctatggcacaaaaatcagttttcaagatgcaacaacagggtgaatataaaccaagaaaggcctttccacaattgccttgggctgaattgaaaaatcctacctacatccaaactaagcatggtaacaagttgttgacatctggtttttggagattcgctagaaagccaaattataccgccgattggattcaagcttgtacatggggtttgaccgctggttttaacactatcatcacaatgtggtaccctatatttttcttggcagttttaatccacagatgtgaaagagattttgctaaatgcgcaagaaagtatggtgacgactgggacgaatactgcaaaactgttaaatggaagttcattccaggtatctattaa |
| DHCR24 from  *Trypanosoma grayi* (*Tg_*DHCR24) | atgactacaaaatccagaagagcatcttcaagatccagaagtaaaaccccagataatggtagtagacaagctaagagattggcacctgaagaattgtacaacttaacagttgaaagaaagtttactccagaaaaggatacttgggacggtcattacgaattctgtggttggatgggtgctttgggtattatgttagcatctcacgtcttgatctattacttttgggtatgcatcgaaaacttccaaggtacattgatctatccaggtcatcctttgttacaaggcgaatcaatgataaccgtttttggtaactacttaagagctcacgctgcaccaacatggggtaccttcggcatgttcactgctttcttgttggttgaatacacattggcagttgtcttaccttctgtagaagttaaaggtttgccaataccttcagaaaacggttacagacaattgtacaagtgtaacgcagttcaagcctggtattgcatgttgttaatcgtcggtgtattccattacactgaaatcttcccattgtggaaattaagagccgattttggtagatatttgactgtcgctacaatttgggccgacgctatatcattgggtgtatacgtagttggtttaagaaagcaaatcagaatgtctcacaacatcatctatgactttttcatgggttcagcattgaactacagattacctggtggtgttgatgtcaaattgtttgccgaatgtagaaactcctgggtattgttgatgatcttgaccttgagtaacgccgctgcaatgcaacatgaaatcggttacgttactggtaacatgtggttcatcgtcgtagcccaatctttgtacgtaaacgctatccaaaagggtgaagaatgtgttatcaccacttgggatttgttttatgaaaagttcggttggatgttagcttactggaatacttgcggtgttccatttttgtactctttacaaggtttctacatacaaacagtcttgaaagacagagaacataagccttggcaattggccttgatgtacgctgtattgatcgttgcatactacatctgggataatgccaactcccaaaagaatagattcagaatgaagagaaacggtacaccacaatccatcttgagaagaaaaagtttcccacaattaccttggggttatattgaaaatcctagagttgtcaagtcagaacgtggtgaattgtttgtcgatggttggtacagatacgcaagaaagttgcattacacagcagacatcataatggccaccttatggggtgtttcttgtggtttcgattcattcataccatttttctacgtttgctttttcttttctcacttagtcgatagagaagctagagacgaatacagatgtagaagaaagtacggtgaattgtgggatagatacatcgaattggttccatacaagtttatacctggtatctattaa |

**Table S3** Plasmids used in this study.

| **Plasmids** | **Description** | **Source** |
| --- | --- | --- |
| pUC57-Kan | Blunt cloning vector, resistant to kanamycin | GenScript |
| pRS425K | *S. cerevisiae* multiple copy plasmid with *LEU2* and *KanMX* marker | This Lab |
| pSW01 | Plasmid with codon optimized DHCR24 from *Homo sapiens* (*Hs*_DHCR24) | This Lab |
| pUC57-Kan-03 | DHCR24 from *Mus musculus* (*Mm*_DHCR24) was codon optimized, synthesized and cloned into pUC57-Kan | This study |
| pUC57-Kan-06 | DHCR24 from zebra fish *Danio rerio* (*Dr*_DHCR24) was codon optimized, synthesized and cloned into pUC57-Kan | This study |
| pUC57-Kan-08 | DHCR24 from *Equus caballus* (*Ec*_DHCR24) was codon optimized, synthesized and cloned into pUC57-Kan | This study |
| pUC57-Kan-09 | DHCR24 from *Gallus gallus* (*Gg*_DHCR24) was codon optimized, synthesized and cloned into pUC57-Kan | This study |
| pUC57-Kan-010 | DHCR24 from *Xenopus tropicalis* (*Xt*_DHCR24) was codon optimized, synthesized and cloned into pUC57-Kan | This study |
| pUC57-Kan-011 | DHCR24 from *Bos taurus* (*Bt*_DHCR24) was codon optimized, synthesized and cloned into pUC57-Kan | This study |
| pRS425k-G01-03 | The cassette T_FBA1_-P_GAL7_-*Mm*_*DHCR24*-T_PGK1_ was cloned and inserted into pRS425k | This study |
| pRS425k-G01-06 | The cassette T_FBA1_-P_GAL7_-*Dr_DHCR24*-T_PGK1_ was cloned and inserted into pRS425k | This study |
| pRS425k-G01-08 | The cassette T_FBA1_-P_GAL7_-*Ec*_*DHCR24*-T_PGK1_ was cloned and inserted into pRS425k | This study |
| pRS425k-G01-09 | The cassette T_FBA1_-P_GAL7_-*Gg*_*DHCR24*-T_PGK1_ was cloned and inserted into pRS425k | This study |
| pRS425k-G01-10 | The cassette T_FBA1_-P_GAL7_-*Xt*_*DHCR24*-T_PGK1_ was cloned and inserted into pRS425k | This study |
| pRS425k-G01-11 | The cassette T_FBA1_-P_GAL7_-*Bt*_DHCR24-T_PGK1_ was cloned and inserted into pRS425k | This study |
| pCRCT-ERG5 | *ERG5* CRISPR knockout plasmid | This lab |
| pRS425k-WRZ1 | The cassette T_FBA1_-P_GAL7_-T_PGK1_ was assembled and inserted into the PstI/BamHI site of pRS425K | This study |
| pRS425k-GXJA1 | The cassette T_TDH2_-P_GAL1_-T_CYC1_ was assembled and inserted into pRS425K | This study |
| pRS425k-GXJA2 | The cassette T_TDH2_-P_GAL7_-T_CYC1_ was assembled and inserted into pRS425K | This study |
| pRS425k-GXJL1 | The cassette *GAL80*_F-*LEU2*-T_FBA1_ was assembled and inserted into pRS425k | This study |
| pRS425k-GXJR1 | The cassette T_PGK1-_*GAL80*_R was assembled and inserted into pRS425k | This study |
| pRS425k-GXJL2 | The cassette *GAL7,10,1_*F*-URA3-*T_TDH2_ was assembled and inserted into pRS425k | This study |
| pRS425k-GXJR2 | The cassette T_CYC1_*_-_GAL7,10,1_*R was assembled and inserted into pRS425k | This study |
| pRS425k-GXJL3 | The cassette *ERG6_*F*-LEU2-*T*_TDH2_* was assembled and inserted into pRS425k | This study |
| pRS425k-GXJR3 | The cassette T_CYC1_*-ERG6_*R was assembled and inserted into pRS425k | This study |
| pRS425k-GXJD1 | The cassette *ERG6_*F*-LEU2-ERG6*_R was assembled and inserted into pRS425k | This study |
| pRS425k-GXJD2 | The cassette *FLD1_*F*-URA3-FLD1*_R was assembled and inserted into pRS425k | This study |
| pRS425k-GXJD3 | The cassette *NEM1_*F*-URA3-NEM1*_R was assembled and inserted into pRS425k | This study |
| pRS425k-GH-01 | The cassette T_FBA1_-P_GAL7_-*Cg*_*DHCR24-6his*-T_PGK1_ was cloned and inserted into pRS425k | This study |
| pRS425k-GH-02 | The cassette T_FBA1_-P_GAL7_-*Tg*_*DHCR24-6his*-T_PGK1_ was cloned and inserted into pRS425k | This study |
| pRS425k-GH-03 | The cassette T_FBA1_-P_GAL7_-*Mm*_*DHCR24-6his*-T_PGK1_ was cloned and inserted into pRS425k | This study |
| pRS425k-GH-05 | The cassette T_FBA1_-P_GAL7_-*At*_*DHCR24-6his*-T_PGK1_ was cloned and inserted into pRS425k | This study |
| pRS425k-GH-07 | The cassette T_FBA1_-P_GAL7_-*Dr_DHCR24*-6his-T_PGK1_ was cloned and inserted into pRS425k | This study |
| pRS425k-GH-09 | The cassette T_FBA1_-P_GAL7_-*Gh*_*DHCR24*-6his-T_PGK1_ was cloned and inserted into pRS425k | This study |
| pRS425k-GH-10 | The cassette T_FBA1_-P_GAL7_-*Ec*_*DHCR24*-6his-T_PGK1_ was cloned and inserted into pRS425k | This study |
| pRS425k-GH-11 | The cassette T_FBA1_-P_GAL7_-*Gg*_*DHCR24*-6his-T_PGK1_ was cloned and inserted into pRS425k | This study |
| pRS425k-GH-12 | The cassette T_FBA1_-P_GAL7_-*Xt*_*DHCR24*-6his-T_PGK1_ was cloned and inserted into pRS425k | This study |
| pRS425k-GH-13 | The cassette T_FBA1_-P_GAL7_-*Bt*_DHCR24-6his-T_PGK1_ was cloned and inserted into pRS425k | This study |
| pRS425k-GH-50 | The cassette T_FBA1_-P_GAL7_-*Hs*_*DHCR24*-6his-T_PGK1_ was cloned and inserted into pRS425k | This study |


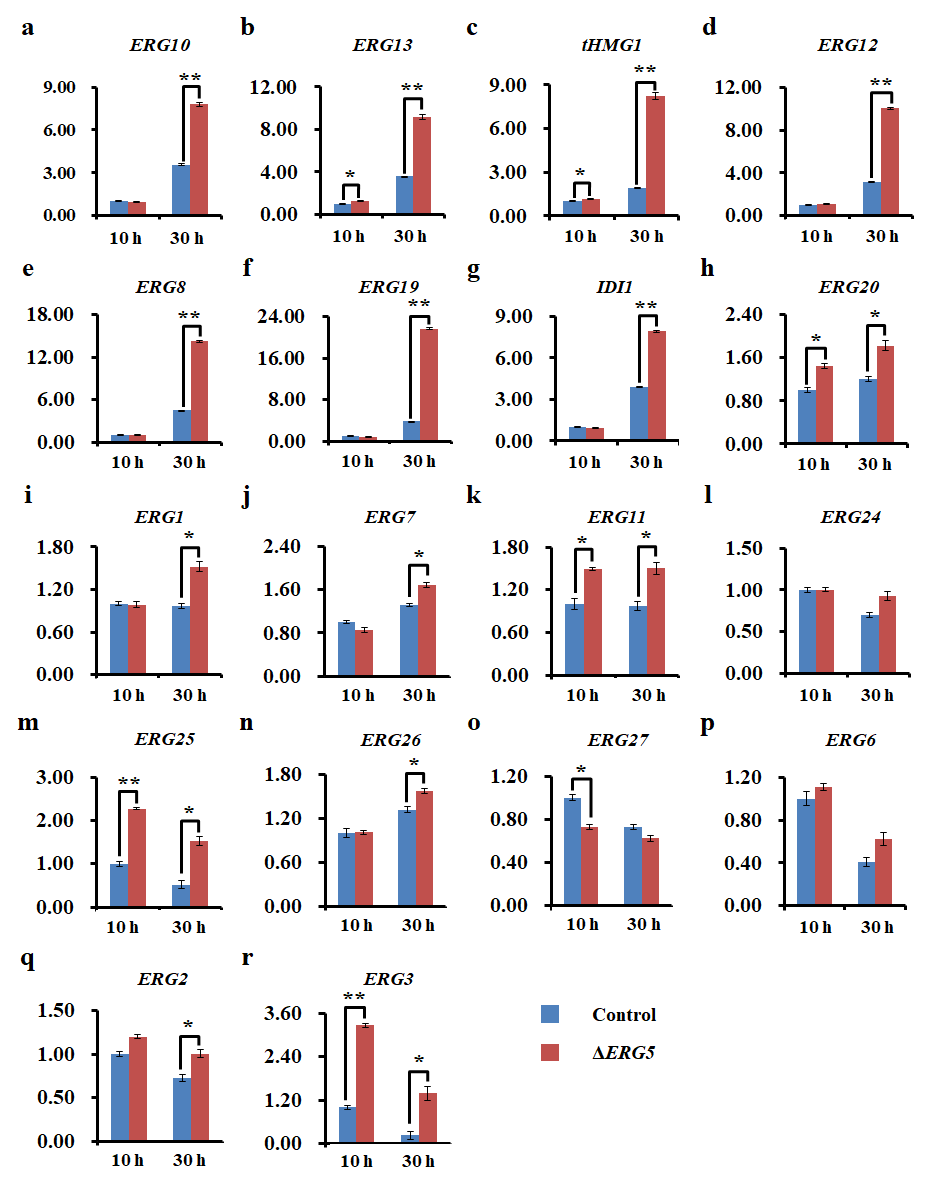


**Figure S1.** Relative transcription level of the MVA pathway genes (a-h) and the post squalene pathway genes (i-r) in control and Δ*ERG5* strain. Cells were harvested at 10 h (glucose consumption phase) and 30 h (ethanol consumption phase). The relative transcription level for each gene was determined as 2^−ΔΔCt^ using gene ALG9 for normalization. All data were from at least triplicate experiments. Significance levels of t-test were determined as “*” is for P< 0.05 and “**” is for P< 0.01.

**
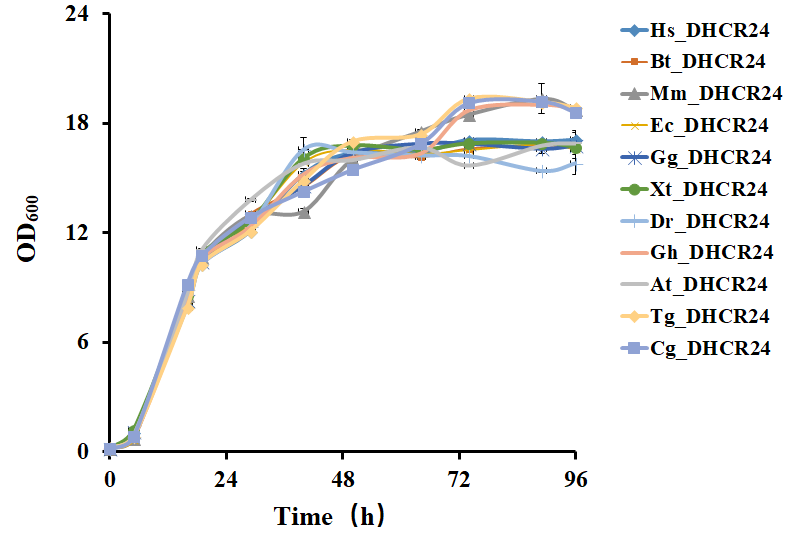
**

**Figure S2.** Cell growths of strains with DHCR24s from diversity species in YPD medium. The error bars represent standard deviation calculated from triplicate experiments. Hs, *Homo sapiens*; Mm, *Mus musculus*; Dr, *Danio rerio*; Ec, *Equus caballus*; Gg, *Gallus gallus*; Xt, *Xenopus tropicalis*; Bt, *Bos Taurus*; At, *Arabidopsis thaliana*; Gh, *Gossypium hirsutum*; Cg, *Cryptococcus gattii*; Tg, *Trypanosoma grayi*.


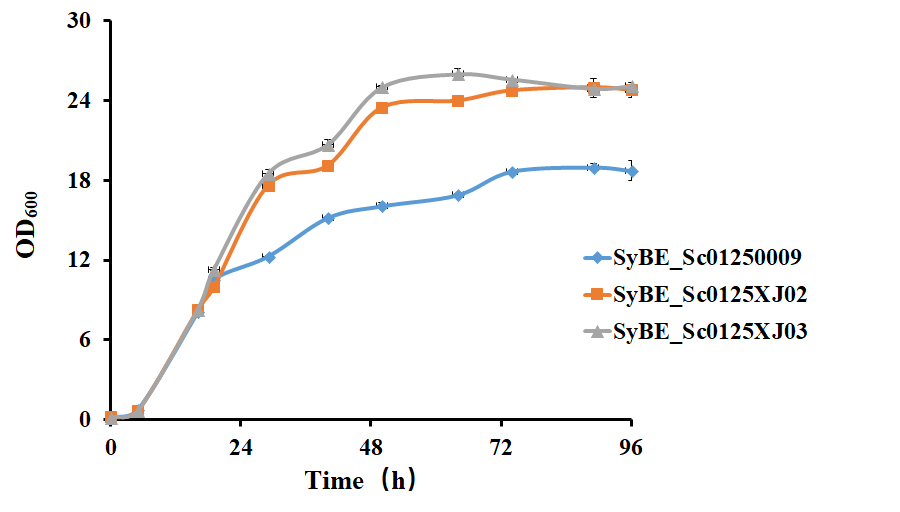


**Figure S3.** Cell growths of strains SyBE_Sc01250009, SyBE_Sc0125XJ02 and SyBE_Sc0125XJ03 in YPD medium.


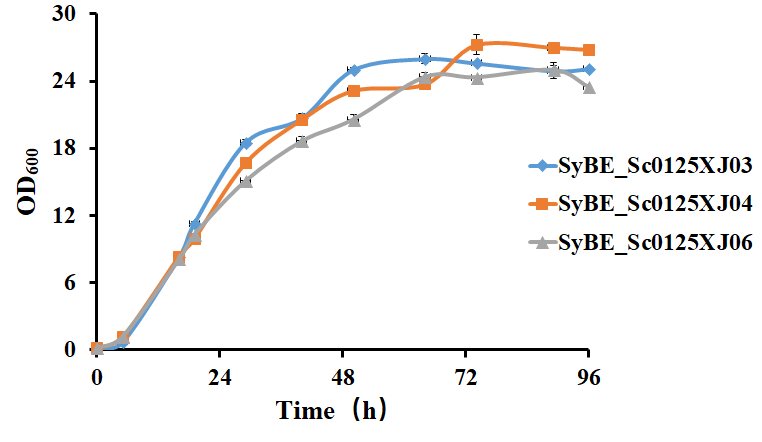


**Figure S4.** Cell growths of strains SyBE_Sc0125XJ03, SyBE_Sc0125XJ04 and SyBE_Sc0125XJ06 in YPD medium.

**
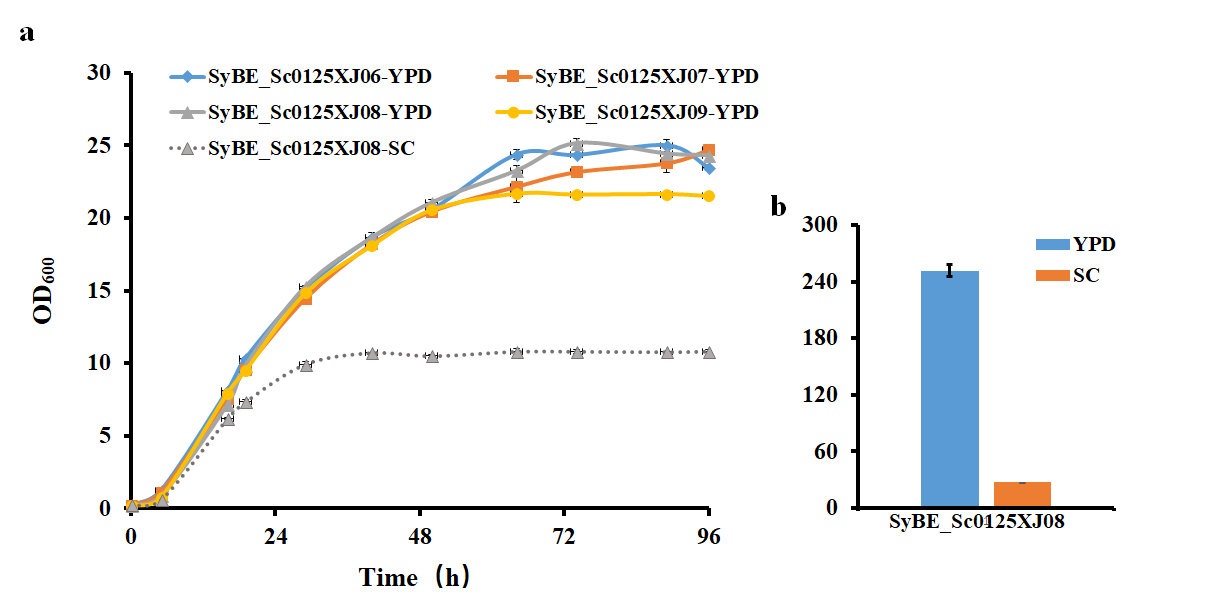
**

**Figure S5. Effect of deleting lipids metabolism associated gene(s) on biomass building-up and 7-DHC production. a** Cell growths of strains SyBE_Sc0125XJ06, SyBE_Sc0125XJ07, SyBE_Sc0125XJ08 and SyBE_Sc0125XJ09 in YPD medium as well as that of SyBE_Sc0125XJ08 in SC medium. **b** 7-DHC production of strain SyBE_Sc0125XJ08 in YPD medium and SC medium.
